# The level of Cry1Ac endotoxin and its efficacy against *H. armigera* in Bt cotton at large scale in Pakistan

**DOI:** 10.1080/21645698.2020.1799644

**Published:** 2020-08-06

**Authors:** Shakra Jamil, Rahil Shahzad, Sajid Ur Rahman, Muhammad Zaffar Iqbal, Muhammad Yaseen, Shakeel Ahmad, Rida Fatima

**Affiliations:** a Genetically Modified Organism Testing Lab, Agricultural Biotechnology Research Institute, Ayub Agricultural Research Institute, Faisalabad, Pakistan; b Department of Mathematics & Statistics, University of Agriculture Faisalabad, Faisalabad, Pakistan; c State Key Laboratory of Rice Biology, China National Rice Research Institute, Hangzhou, China

**Keywords:** Canopies, cry endotoxin, ELISA, insect mortality, insect resistance, strip test, transgenic cotton

## Abstract

A biophysical survey was conducted in 15 cotton-growing districts of Pakistan. Four hundred cotton growers were approached and inquired about the production technology of Bt cotton. Further, 25 strip tests using combo strips (*Cry1Ac, Cry2Ab, Vip3Aa* and *Cp4, EPSPS* gene) were performed at each farmer’s field. Out of 10,000 total-tested samples, farmers claimed 9682 samples as Bt and 318 samples as non-Bt. After performing a strip test, 1009 and 87 samples were found false negative and false positive, respectively. Only 53 samples were found positive for *Cry2Ab*, 214 for *EPSPS* and none for *Vip3Aa* gene. Quantification of Cry endotoxin and bioassay studies were performed by taking leaves from upper, middle, and lower canopies, and fruiting parts at approximately 80 days after sowing from 89 varieties. Expression was highly variable among different canopies and fruiting parts. Moreover, Cry endotoxin expression and insect mortality varied significantly among varieties from 0.26 µg g^−1^ to 3.54 µg g^−1^ with mortality ranging from 28 to 97%, respectively. Highest *Cry1Ac* expression (3.54 µg g^−1^) and insect mortality (97%) were observed for variety FH-142 from DG Khan. Cry endotoxin expression varied significantly across various plant parts, i.e., IUB-13 variety from upper canopy documented 0.34 µg g^−1^ expression with 37% insect mortality in Layyah to 3.42 µg g^−1^ expression and 96% insect mortality from DG Khan. Lethal dose, LD95 (2.20 µg g^−1^) of Cry1Ac endotoxin was optimized for effective control of *H. armigera*. Our results provided evidence of practical resistance in *H. armigera* and way forward.

## 1. Introduction

The *Helicoverpa armigera* remained the historical insect pest of Pakistan which brutally damaged cotton crop during 1990s.^1^ At that time, usage of pesticides having carcinogenic and neurotoxic affect was common.^2^ Development of genetically modified (GM) cotton, expressing insect resistance Cry proteins, provided an alternate safe option for the control of American bollworm.^3^ Less production cost, high yield and more profit are the main benefits provided by the GM cotton.^4,5^ These benefits urged farmers for the cultivation of Bt cotton for the first time in 2000. On the basis of farmers’ acceptability and success stories of Bt cotton, it was officially approved for general cultivation by National Biosafety Committee (NBC) of Pakistan during 2010.^6^

Cry proteins are a large family of crystalline toxins produced by Bacillus thuringiensis. Individually, the family members are highly specific, but collectively, they target a diverse range of insects and nematodes.^7^ Insect control depends mainly on concentration of Cry protein ingested, larvae age and time of exposure.^8^ However, the continuous success of Bt cotton has been challenged by evolution of pest resistance.^9^ Certain countries have reported resistance in Pink Bollworm against Cry1Ac gene.^10^ Similarly, various studies from USA,^11^ India,^12^ Argentina,^13^ South Africa^14^ and Brazil^15^ have reported resistance in seven major insect species excluding *H. armigera* against different Cry proteins including Cry1Ac in cotton and corn. Resistance of *H. armigera* against *Cry1Ac* has been observed in few countries like China.^16^

The possible reasons for development of resistance in bollworms against Cry proteins are, i.e., variable expression in different plant parts, inconsistent promotor activity, and cultivation of unapproved varieties with sub-lethal levels of Cry endotoxin.^6,17^ For sustainable pest control, cotton plants should produce Bt toxin at or above lethal levels to control insects. Moreover, all plant parts should uniformly produce Bt toxin at critical period of insect attack. Many studies have proved that accumulation of Bt toxin is highly variable and depends on genotype, age of plant, environment and plant tissues.^17,18^

Special attention is being paid to critical expression level, defined as the minimum protein expression level to control target pest. It is influenced by different geographical conditions due to variability in adaptation mechanism of insect pest.^19^ There is a dire need to re-optimize lethal dose (LD95) of Cry1Ac against *H. armigera*. Previously, it was reported that variable LD95, i.e.,, 1.62 µg g^−1^, 2.04 µg g^−1^, 1.90 µg g^−1^, 335.7 µg ml^−1^ and 0.09 to 9.07 µg ml^−1^ was found in different countries, i.e., Australia, Spain, India, Pakistan, and China, respectively.^17^ Earlier, in Pakistan, two studies have been conducted in which they quantify the expression of Cry1Ac protein and determine its lethal level by using fresh cotton leaf tissues. An experiment conducted in 2014 reported LD95 value of 770 + 25 ng g^−119^ whereas second study conducted during 2016 but published in 2019 reported 1.59 µg g^−117^ lethal level of Cry1Ac for effective control of *H. armigera*. However, to the best of my knowledge, no comprehensive study regarding field survey at farmer level of core cotton areas has been conducted.

Therefore, this study was conducted for detection, identification, and quantification of Bt cotton (Cry1Ac event Mon531) in approximately all cotton-growing districts of Punjab (15 districts) (Table S1). In addition to that, status of *H. armigera* resistance against Bt cotton was assessed via insect bioassays using fresh leaves from survey places at 80 DAS. Furthermore, the status of new Bt genes i.e., *Cry2Ab, Vip3A* and herbicide-tolerant *Cp4, EPSPS* gene was also assessed using combo immunostrip assay.

## 2. Materials and methods

### 2.1. Experimental site

**Figure 1.**
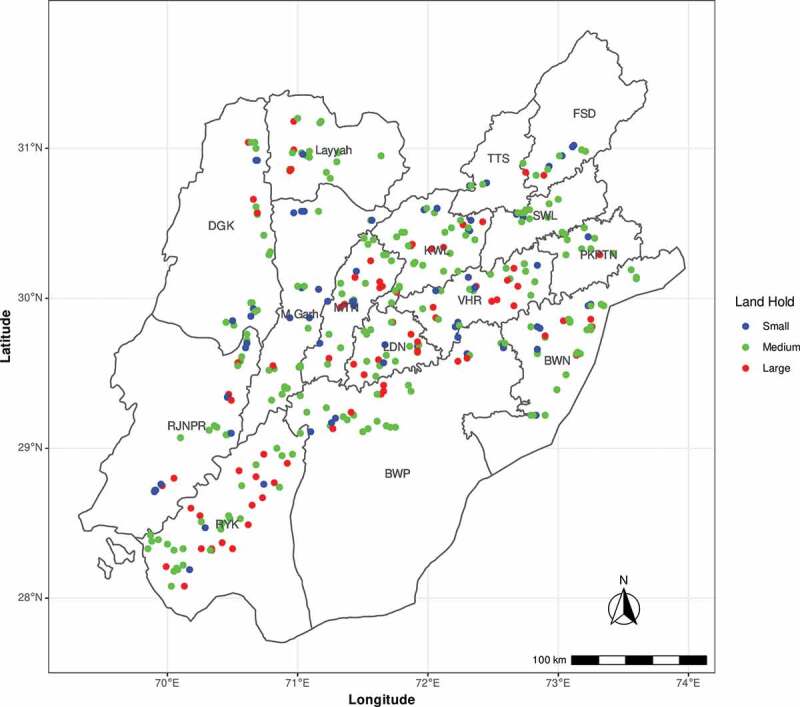
Punjab Map showing geographical display of farmers covered in survey representing small, medium and low land farmers from fifteen cotton-growing districts with respect to their GPS coordinates. Whereas FSD denotes Faisalabad, TTS (Toba Tek Singh), SWL (Sahiwal), PKPTN (Pakpattan), VHR (Vehari), KWL (Khanewal), BWN (Bahawalnagar), BWP (Bhawalpur), LDN (Lodhran), MTN (Multan), RYK (Rahim Yar Khan), M. Garh (Muzaffargarh), DGK (DG Khan) and RJNPR (Rajanpur).

The present study was conducted in Punjab, Pakistan to observe purity of Bt cotton seed with respect to insect-resistance genes *Cry1Ac* (event MON531), *Cry2Ab* (MON15985), *Vip3Aa* (COT102) and herbicide tolerant *Cp4, EPSPS* gene (MON1445). Total 400 farmers were surveyed from five cotton-growing divisions, (15 districts, 47 tehsils (Administrative Units) and 400 mauzas) after obtaining information from Director General Agriculture Extension, Punjab about cotton production plan (Table S1). Latitude and longitude information was recorded from each farmer’s location as provided in Table S2. The information about farmers were classified to three major categories based on land owned by them, i.e., (Small (0–10 acres), Medium (11–50 acres) and Large (51 or above acres)) and survey represented each category from all districts with majority of farmers from medium category (Figure 1).

### 2.2. Survey questionnaire and strip test

Questionnaire-based information was collected from each farmer about education, total land, and inputs used. . Twenty-five strip tests were performed at each farmer’s field irrespective of cotton varieties cultivated using QuickStix Combo Kits (EnviroLogix) for identification of three insect-resistance Bt genes and one herbicide-tolerant *Cp4, EPSPS* gene. Two leaf punch samples (approximately 10 mg each) were taken by snapping cap of disposable Eppendorf tube down on the leaf. The leaf tissues were ground by rotating disposable pestle against walls of tubes by twisting motion and process continued until fine grinding. 0.5 ml of 1X extraction buffer was added to the tube and leaf tissue was thoroughly mixed with extraction buffer. QuickStix Combo strips were dipped in leaf extract and examined after 10 minutes until appearance of final bands on strip and results were recorded. Tested 20 strips of each category showing results for Non-Bt (Fig S1a) Cry1Ac alone (Fig S1b), *Cp4, EPSPS* alone (Fig S1 c), Vip3Aa alone (Fig S1d), Cry1Ac and RR gene in combination (Fig S1e) and Cry1Ac, Cry2Ab and *Cp4, EPSPS* genes in combination (Fig S1 f) are provided to increase reviewers confidence.

### 2.3. Quantitative ELISA

Six top cultivated varieties were selected from each district for quantification of Cry proteins. Five healthy plants (biological repeats) were selected from each variety and 3 leaves of same color, size and age from upper, middle, and lower canopy as well as 3, 10 days old bolls and 3 squares at approximately 80 days after sowing (DAS) of 89 varieties from 15 cotton-growing districts. Six top cultivating varieties were selected from each district except 05 from Toba Tek Singh because the surveyed farmers from their has only cultivated 05 varieties. Enzyme-Linked Immunosorbent Assay (ELISA) was used for quantification of Cry1Ac proteins. The whole procedure was repeated for making of plant extract as described for lateral flow strip test. Additionally, plant weight was also recorded (approximately 20 mg) separately for upper, middle, and lower canopy as well as 10 days old bolls and squares. Quantification of Cry1Ac was done through ELISA following the instruction provided by vendor (EnviroLogix) with ELISA kit as described in supplementary file section 1.1.

### 2.4. *Detached leaf bioassay for* H. armigera

*H. armigera* larvae were collected from okra and cotton fields at Ayub Agricultural Research Institute, Faisalabad and reared up to pupation on chickpea-based artificial diet in plastic cups (5 m diameter × 6 m height) individually.^17,20^ Moths were kept in glass cages (30 × 30 × 30 cm) after emergence at 25 ± 2oC and 70 ± 5% R.H. Adults were fed on 10% honey solution and small pieces of muslin cloth were hung inside the box for oviposition of adults.^21^ First instar larvae after hatching were shifted in petri plates and were kept on artificial diet until development of 2^nd^ instar insects. First instar larvae although are more harmful as compared to 2*nd* instar but were very delicate and difficult to handle hence 2^nd^ instar larvae were used for bioassay studies.

For each variety, leaves were from three plants positions: upper canopy (1/3 from top), middle canopy (1/2 plant height) and lower canopy (1/3 from plant base) in triplicate from transgenic (88) and non-transgenic (01) varieties. Similarly, 10 days old bolls and squares were also gathered in triplicate from each plant. After taking punches for ELISA to quantify Cry proteins, the petiole of each leaf was wrapped in wet tissue and placed in glass petri plates (6” diameter). Weights were recorded for five 2nd instar larvae and placed them in petri plates for bioassay. Petri plates were then wrapped by para-film sheet to avoid escape of larvae. Bioassay was conducted for 96 h and later on insect mortality% age was recorded for each treatment/variety. Controlled environmental conditions were provided, i.e., 26 ± 3ºC temperature, 70 ± 10% relative humidity and 16 h photoperiod throughout the bioassay period. Bioassay for 10 days cotton bolls and cotton squares were performed using method described by^22^ as described in supplementary material section 1.2.

### 2.5. Statistical data analysis

Analysis of Variance (ANOVA) was performed for comparing varieties in terms of leaf damage%, Bt toxin concentration, and larvae mortality%. Tukey’s Honest Significant Difference test was used to conduct multiple mean comparisons beyond ANOVA. Linear model was done for characterization and quantification of various inputs, i.e., irrigation, rainfall, nitrogen, phosphorus, potassium, and calcium on Bt expression.^23^ Logistic regression analysis was used for the determination of *H. armigera* mortality against Cry1Ac endotoxin expression and to calculate lethal doses i.e. LD50, LD90 and LD95 of Cry toxin for control of *H. armigera*.^24^ Further, spatial analysis of farmers was performed on the basis of landholdings, i.e., small (blue), medium (yellow) and high (orange). Farmers were plotted on Punjab map to show the frequency and distribution of each category of farmers covered in the survey.^25^ Distributions analysis of Bt and non-Bt cotton with reference to Cry1Ac, Cry2Ab and Vip3Aa gene and herbicide-tolerant *Cp4, EPSPS* gene in all surveyed districts was performed. Rainfall was classified into three categories such as low (below 3 rain showers), normal (below 5 rain showers), high (5, or above rain showers) for data analysis. All analysis were performed using R-3.6.1

## 3. Results

### 3.1. Farmer’s perception vs diagnostic test of bt cotton housing Cry1Ac gene

A total of 400 farmers (Table S2) were surveyed and 25 samples were tested through lateral flow strip test from each farmer. Collectively 10,000 cotton samples in aggregate were tested from all over Punjab for four genes, i.e., 03 Bt genes (*Cry1Ac, Cry2Ab* and *Vip3Aa*) and 01 herbicide-tolerant *Cp4, EPSPS* gene. The surveyed farmers belong to three categories: small (1–10 acres), medium (11–50 acres) and large farmers (above 50 acres) Figure 1. Among 10,000 samples, 9682 and 318 cotton samples were reported as Bt and non-Bt, respectively, by farmers. Whereas, out of 9682 Bt samples, 10% samples (1009) were found false negative (Type I error) for *Cry1Ac* gene. Similarly, among 318 non-Bt samples, 27% samples (81) were found false positive (Type II error). Overall, 12% cotton samples (1240) and 88% cotton samples (8760) were found negative and positive for Cry1Ac, respectively (Table 1).

**Table 1.** Status of Cry1Ac gene in Punjab Pakistan (farmer’s perception Vs diagnostic test).

Farmers perception (Bt samples)				
		Cry1Ac gene		
Division	District	Negative	Positive	Total
Bahawalpur	Bahawalpur	64 (7%)	831 (93%)	895
	Bahawalnagar	117 (13%)	799 (87%)	916
	Rahim Yar Khan	125 (12%)	937 (88%)	1062
	Total	306 (11%)	2567 (89%)	2873
Multan	Multan	32 (4%)	694 (96%)	726
	Lodhran	26 (4%)	574 (96%)	600
	Khanewal	46 (4%)	1000 (96%)	1046
	Vehari	60 (7%)	790 (93%)	850
	Total	164 (5%)	3058 (95%)	3222
DG Khan	DG Khan	133 (24%)	430 (76%)	563
	Layyah	53 (11%)	440 (89%)	493
	Muzaffargarh	207 (23%)	693 (77%)	900
	Rajanpur	87 (14%)	544 (86%)	631
	Total	480 (19%)	2107 (81%)	2587
Sahiwal	Sahiwal	17 (5%)	333 (95%)	350
	Pakpattan	0 (0%)	250 (100%)	250
	Total	17 (3%)	583 (97%)	600
Faisalabad	Faisalabad	42 (14%)	258 (86%)	300
	Toba Tek Singh	0	100 (100%)	100
	Total	42 (10%)	358 (90%)	400
	Total Bt samples	1009 (10%)	8673 (90%)	9682
Farmers perception (Non-Bt samples)				
Division	District	Cry1Ac gene		Total
		Negative	Positive	
Bahawalpur	Bahawalpur	02 (40%)	03 (60%)	5
	Bahawalnagar	26 (76%)	8 (24%)	34
	Rahim Yar Khan	76 (86%)	12 (14%)	88
	Total	104 (82%)	23 (18%)	127
Multan	Multan	19 (79%)	5 (21%)	24
	Lodhran	0	0	0
	Khanewal	4 (100%)	0 (0%)	4
	Vehari	0	0	0
	Total	23 (82%)	5 (18%)	28
DG Khan	DG Khan	63 (72%)	24 (28%)	87
	Layyah	7 (100%)	0 (0%)	7
	Muzaffargarh	0	0	0
	Rajanpur	34 (49%)	35 (51%)	69
	Total	104 (64%)	59 (36%)	163
Sahiwal	Sahiwal	0	0	0
	Pakpattan	0	0	0
	Total	0	0	0
Faisalabad	Faisalabad	0	0	0
	Toba Tek Singh	0	0	0
	Total	0	0	0
	Total of Bt samples	231 (73%)	87 (27%)	318
	Grand Total	1240 (12%)	8760 (88%)	10,000

Collectively, 3000 cotton samples were tested from the Bahawalpur division. Out of this, 2873 samples were claimed Bt by farmers, whereas, 127 samples were claimed not-Bt. A total of 306 samples (11%) were found false negative from Bt samples. Similarly, 23 samples (18%) were observed false positive for *Cry1Ac* from non-Bt samples. From Multan division (3250) cotton samples were tested, From which 3222 were claimed Bt by farmers, while 28 samples were claimed non-Bt. Diagnostic testing revealed that among 3222 cotton samples, 164 samples (5%) were found false negative, whereas among 28 non-Bt samples, 05 samples (18%) were found false positive for Cry1Ac gene (Table 1). From DG Khan Division, 2750 cotton samples were tested. Out of these, 2587 samples were believed Bt by farmers and 163 samples were believed non-Bt. Diagnostic testing found that 480 samples (19%) were false negative for Cry1Ac gene. Likewise, 59 samples (36%) were false positive for Cry1Ac gene. No samples from Sahiwal and Faisalabad divisions were claimed non-Bt, hence no false positive results were observed. Six hundred samples were tested under Bt category from Sahiwal division. Out of 600, 3% (17 samples) were observed false negative for *Cry1Ac* gene. Only 400 cotton samples were tested for *Cry1Ac* gene from Faisalabad division. 358 samples (90%) of them were found positive, whereas 42 samples (10%) were found false negative for *Cry1Ac* gene (Table 1).

### 3.2. Status of Cry2Ab, Vip3Aa and Cp4, EPSPS gene in Punjab

Collectively, 53 samples (0.53%) were found positive for Cry2Ab gene from Bahawalpur, Multan, and DG Khan Division and none from Sahiwal and Faisalabad division. Highest number of Cry2Ab positive samples were found from Multan division (43 samples) followed by DG Khan (06 samples) and Bahawalpur (04 samples). District Multan possessed highest Cry2Ab positive (26) samples followed by Lodhran (10 samples). No sample was found positive for Vip3Aa gene from all over Punjab (Table 2). On the other hand, total 224 samples (2.2%) were found positive for *Cp4, EPSPS* gene. The frequency of positive samples for *Cp4, EPSPS* gene was high (170 samples) in Multan division, whereas no positive samples was observed from Sahiwal division. Similarly, DG Khan, Bahawalpur, and Faisalabad divisions possessed 43, 11, and 04 samples, respectively (Table 2).

**Table 2.** Status of new Bt genes (Cry2Ab and Vip3Aa) and herbicide tolerant (*Cp4, EPSPS*) gene in Punjab Pakistan.

		Cr2Ab gene			Vip3Aa gene			*Cp4, EPSPS* gene		
Division	District	Negative	Positive	Total	Negative	Positive	Total	Negative	Positive	Total
Bahawalpur	Bahawalpur	900 (100%)	0 (0%)	900	900 (100%)	0 (0%)	900	893 (99.2%)	7 (0.8%)	900
	Bahawalnagar	950 (100%)	0 (0%)	950	950 (100%)	0 (0%)	950	950 (100%)	0 (%)	950
	Rahim Yar Khan	1146 (99.6%)	4 (0.4%)	1150	1150 (100%)	0 (0%)	1150	1046 (99.6%)	4 (0.4%)	1150
	Total	2996 (99.8%)	4 (0.2%)	3000	3000 (100%)	0 (0%)	3000	2989 (99.6%)	11 (0.4%)	3000
Multan	Multan	724 (96.5%)	26 (3.5%)	750	750 (100%)	0 (0%)	750	675 (90%)	75 (10%)	750
	Lodhran	590 (98.3%)	10 (1.7%)	600	600 (100%)	0 (0%)	600	581 (96.8%)	19 (3.2%)	600
	Khanewal	1050 (100%)	0 (0%)	1050	1050 (100%)	0 (0%)	1050	1009 (96%)	41 (4%)	1050
	Vehari	843 ((99.2%)	7 (0.8%)	850	850 (100%)	0 (0%)	850	815 (96%)	35 (4%)	850
	Total	3207 (98.7%)	43 (1.3%)	3250	3250 (100%)	0 (0%)	3250	3080 (94.7%)	170 (5.3%)	3250
DG Khan	DG Khan	650 (100%)	0 (0%)	650	650 (100%)	0 (0%)	650	645 (99.2%)	5 (0.8%)	650
	Layyah	494 (98.8%)	6 (1.2%)	500	500 (100%)	0 (0%)	500	482 (96.4%)	18 (3.4%)	500
	Muzaffargarh	900 (100%)	0 (0%)	900	900 (100%)	0 (0%)	900	889 (98.7%)	11 (1.3%)	900
	Rajanpur	700 (100%)	0 (0%)	700	700 (100%)	0 (0%)	700	691 (98.7%)	9 (1.3%)	700
	Total	2744 (99.7%)	6 (0.3%)	2750	2750(100%)	0 (0%)	2750	2707 (98.4%)	43 (1.6%)	2750
Sahiwal	Sahiwal	350 (100%)	0 (0%)	350	350 (100%)	0 (0%)	350	350 (100%)	0 (0%)	350
	Pakpattan	250 (100%)	0 (0%)	250	250 (100%)	0 (0%)	250	250 (100%)	0 (0%)	250
	Total	600 (100%)	0 (0%)	600	600 (100%)	0 (0%)	600	600 (100%)	0 (0%)	600
Faisalabad	Faisalabad	300 (100%)	0 (0%)	300	300 (100%)	0 (0%)	300	288 (100%)	12 (0%)	300
	Toba Tek Singh	100 (100%)	0 (0%)	100	100 (100%)	0 (0%)	100	100 (100%)	0 (0%)	100
	Total	400 (100%)	0 (0%)	400	400 (100%)	0 (0%)	400	400 (100%)	0 (0%)	400
	Grand Total	9947 (99.5%)	53 (0.5%)	10000	10000 (100%)	0 (0%)	10000	9776 (97.8%)	224 (2.2%)	10000

### 3.3. Cry1Ac expression and leaf bio-toxicity assay from different plant parts

Quantification and insect bio-toxicity assay for Cry1Ac protein were performed at different plant parts including leaves, bolls, and squares. One standard non-Bt cultivar NIAB-Kiran from Multan was used as a negative control to check validity of results (Figure 1). Insect bioassays and quantification of Cry1Ac protein from different plant parts were done separately with four biological repeats.

#### 3.3.1. Upper Canopy (UC) expression and insect mortality

**Figure 2.**
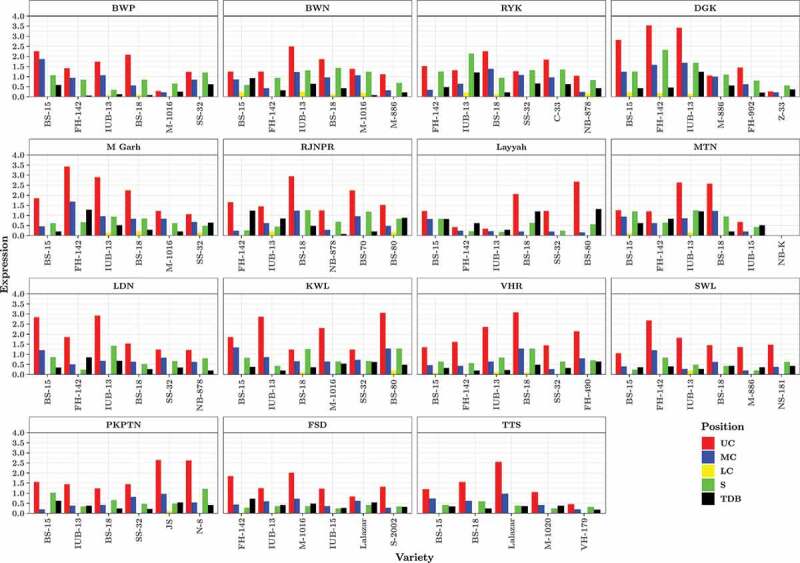
Quantification of Cry1Ac endotoxin from upper, middle & lower canopy leaves and ten days bolls and squares of farmer’s field grown 89 cotton varieties across 15 cotton growing districts of Punjab. Whereas BWP (Bahawalpur), BWN (Bahawalnagar), RYK (Rahim Yar Khan), DGK (DG Khan), M Garh (Muzaffargarh), RJNPR (Rajanpur), MTN (Multan), LDN (Lodhran), KWL (Khanewal), VHR (Vehari), SWL (Sahiwal), PKPTN (Pakpattan), FSD (Faisalabad), TTS (Toba Tek Singh), expression (Cry1Ac expression), UC (Upper Canopy), MC (Middle Canopy), LC (Lower Canopy), S (Squares), TDB (Ten days bolls). Five healthy plants were used for each bioassay study as biological replicate.

**Figure 3.**
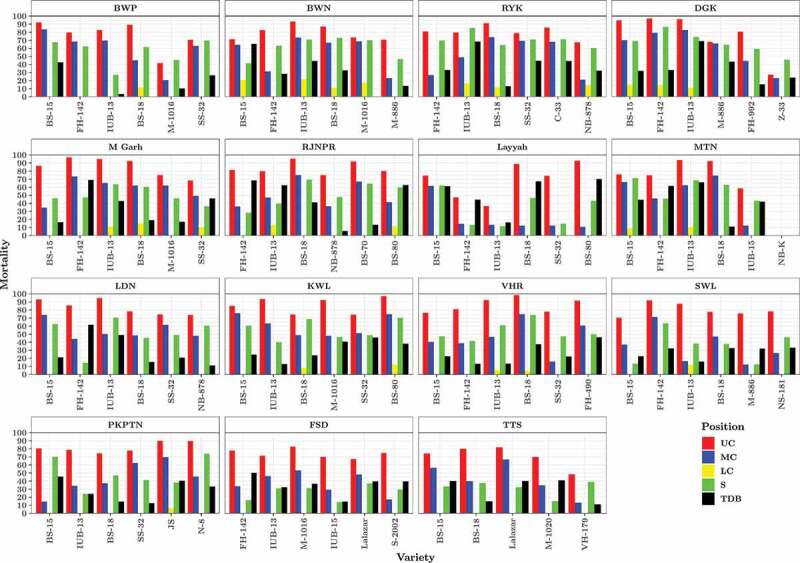
Bio-toxicity assay for *H. armigera* on upper, middle & lower canopy leaves and ten days bolls and squares of farmer’s field grown 89 cotton varieties against Cry1Ac endotoxin across 15 cotton-growing districts of Punjab. Whereas BWP (Bahawalpur), BWN (Bahawalnagar), RYK (Rahim Yar Khan), DGK (DG Khan), M Garh (Muzaffargarh), RJNPR (Rajanpur), MTN (Multan), LDN (Lodhran), KWL (Khanewal), VHR (Vehari), SWL (Sahiwal), PKPTN (Pakpattan), FSD (Faisalabad), TTS (Toba Tek Singh), expression (Cry1Ac expression), UC (Upper Canopy), MC (Middle Canopy), LC (Lower Canopy), S (Squares), TDB (Ten days bolls). Five healthy plants were used for each bioassay study as biological replicate.

267 leaves were collected for estimation of Bt endotoxin from upper canopy. Expression in the UC varied from zero (NIAB-Kiran) to 3.54 µg g^−1^ (FH-142). Only 8 varieties showed expression below 1.0 µg g^−1^ whereas four varieties showed above 3.0 µg g^−1^ expression. FH-142 showed highest expression in DG Khan (3.54 µg g^−1^) and Muzaffargarh districts (3.42 µg g^−1^) followed by Sahiwal (2.68 µg g^−1^). IUB-13 variety showed consistently good expression in almost all districts. Similarly, BS-18 also showed expression at some locations, i.e., Vehari (3.08 µg g^−1^), Rajanpur (2.94 µg g-1), Multan (2.58 µg g-1) and Rahim Yar Khan (2.25 µg g-1) (Figure 2). Collectively, 1335 2^nd^ instar larvae were used for 267 bioassay studies in upper canopy leaves. Larval survival rate was 21% with 1053 larvae dead and 282 were alive after 96 h of feeding. Mortality varied from 0 (NIAB-Kiran, non-Bt variety from Multan) to > 95% from BS-18 (Vehari), BS-80 (Khanewal), FH-142 (DG Khan), FH-142 (Muzaffargarh) and IUB-13 (DG Khan) (Figure 3).

#### 3.3.2. Middle Canopy (MC) expression and insect mortality

Cry1Ac expression in MC varied from 0 to 1.86 µg g^−1^. Seventy-two varieties showed Bt expression below 1.0 µg g^−1^, whereas remaining 17 varieties were above 1.0 µg g^−1^ expression. Highest MC expression was observed for BS-15 (1.86 µg g^−1^) from Bahawalpur followed by FH-142 (1.68 µg g^−1^) from Muzaffargarh (Figure 2). Insect mortality was drastically reduced in MC. Out of 1335 insects used for bioassay studies in MC, 640 were found dead and 695 were alive (48% mortality). Only 8 varieties, i.e., BS-18 (Multan, Vehari), BS-80 (Khanewal), BS-18 (Rajanpur), BS-15 (Khanewal, Bhawalpur), IUB-13 and FH-142 (DG Khan) showed above 75% mortality (Figure 3).

#### 3.3.3. Lower Canopy (LC) expression and insect mortality

Overall expression in the LC varied from 0 to 2.25 µg g^−1^. Sixty-three varieties from LC behaved like non-Bt and showed no expression and remaining 26 varieties showed 0.08 to 0.25 µg g^−1^ expressions. Nine of the positive samples from lower canopy were of IUB-13 from different districts while remaining 17 samples belonged to BS-15, BS-18, MNH-1016 and FH-142 (Figure 2). Insect mortality on LC leaves was very low (3% only). Only 44 insects died and rest 1291 were found alive and healthy. Two varieties from Bahawalnagar, i.e., BS-15 and IUB-13 showed above 20% insect mortality. One ninety five bioassays from 65 varieties did not show any insect mortality. Whereas 72 insect bioassays showed insect mortality varying from 5–20% (Figure 3).

#### 3.3.4. Ten Days Bolls (TDB) expression and insect mortality

Highest Cry1Ac expression (1.32 µg g^−1^) in TDBs was observed for BS-80 from Layyah followed by FH-142 (1.28 µg g^−1^) from Muzaffargarh. Lowest expression (0 µg g^−1^) for TDB was noted for NIAB-Kiran and SS-32 from Multan and Layyah, respectively. Only 7 varieties i.e., IUB-13 (Rahim Yar Khan), BS-18 (Layyah), IUB-13 (Multan), IUB-13 (DG Khan), FH-142 (Rajanpur), FH-142 (Muzaffargarh) and BS-80 (Layyah) showed expression above or equal to 1.0 µg g^−1^ (Figure 2). Exocarp of three TDBs from each variety were used for one bioassay, hence collectively 9 bolls were used for each variety. Insect mortality on TDBs varied from 0 to 70%. Out of 1335 larvae of *H. armigera*, only 433 larvae were found dead and remaining 902 larvae were alive (Figure 3).

#### 3.3.5. Squares expression and insect mortality

*Cry1Ac* expression in squares varied from 0 to 2.32 µg g^−1^. Twenty-eight varieties showed expression between 0 to 0.5 µg g^−1^. While, 38 varieties showed Bt expression from 0.51–1.0 µg g^−1^. Likewise, 20 varieties showed Bt expression 1.1–1.5 µg g-1. Only 2 varieties, i.e., IUB-13 (Rahim Yar Khan) and FH-142 (from DG Khan) recorded above 2.0 µg g-1 expression (Figure 2). Bioassay of squares was performed by placing 3 cotton squares and five 2^nd^ star larvae of *H. armigera* in each petri plate. Out of 1335, 659 and 676 insects were found dead and alive, respectively. Highest insect mortality (87%) was recorded for FH-142 from DG Khan, while lowest insect mortality (0%) was recorded for NIAB-Kiran from Multan. Forty-nine varieties showed below 50% insect mortality and remaining 40 varieties showed 50–87% insect mortality (Figure 3).

### 3.4. Expression profiling of Bt cotton varieties through ELISA and insect bioassay in different districts

**Figure 4.**
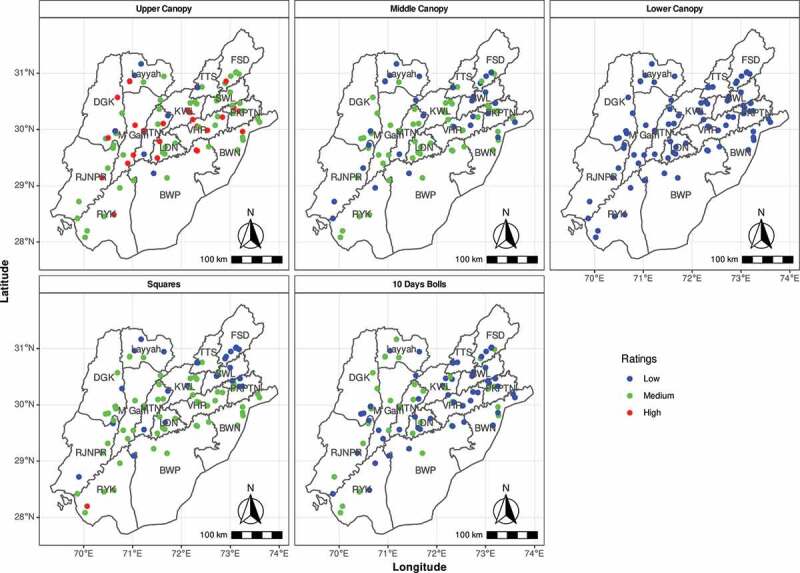
District wise expression profiling of 89 varieties from Upper, Middle, Lower Canopy, 10 days bolls and Squares from 15 cotton-growing districts displayed according to GPS Coordinates of the farmers. Whereas Low indicates (0–0.5 µg g-1), Medium (0.6–2.0 µg g-1) and High (2.1-Above µg g-1). Whereas BWP (Bahawalpur), BWN (Bahawalnagar), RYK (Rahim Yar Khan), DGK (DG Khan), M Garh (Muzaffargarh), RJNPR (Rajanpur), MTN (Multan), LDN (Lodhran), KWL (Khanewal), VHR (Vehari), SWL (Sahiwal), PKPTN (Pakpattan), FSD (Faisalabad), TTS (Toba Tek Singh), expression (Cry1Ac expression), UC (Upper Canopy), MC (Middle Canopy), LC (Lower Canopy), S (Squares), TDB (Ten days bolls). Five healthy plants were used for each bioassay study as biological replicate.

Spatial display of Bt concentration was developed for 6 locations from each district by ranking Bt expression as low (0–0.5 µg g-1), medium (0.6–2.0 µg g-1) and high (> 2.0 µg g-1) using blue, yellow and orange colors dots, respectively (Figure 4). UC expression from 30 locations was marked high, while medium for 55 locations and low from 6 locations. Likewise, 33 locations reported low expression, 56 showed medium expressions and high expression was not observed from MC. Expression in LC was low in all 89 locations. Similarly, TDBs also marked low expression from 57 locations while 32 locations showed medium expression. Relatively higher expression was found in squares. Only 28 locations recorded low expression, 59 showing medium expression and two showed high expression (Figure 4). District Muzaffargarh showed highest average *Cry1Ac* expression and insect mortality. Lowest average expression and insect mortality was observed in Layyah (Table S3).

Highest average expression in MC was observed in DG Khan with 61% insect mortality. Layyah recorded lowest average MC *Cry1Ac* expression with 21% insect mortality (Table S4). Average expression in the LC was negligible in all districts except with 12% insect mortality (Table S5). *Cry1Ac* expression in TDB was relatively higher in all districts. Highest TDBs expression was recorded in Layyah with 43% insect mortality. Lowest expression in TDB was recorded from Bahawalpur (Table S6). Highest Cry1Ac expression in squares (1.30 µg g-1) was observed from Rahim Yar Khan with 70% insect mortality whereas lowest expression was observed from Faisalabad (0.30 µg g-1) with 26% insect mortality (Table S7). Almost all plant position from non-core cotton districts recorded low Cry1Ac expression as compared to core cotton-producing districts.

### 3.5. Expression profiling of bt cotton varieties for Cry1Ac gene through ELISA and insect bioassay

Due to repeated cultivation in more than one district, all 89 locations from 15 districts summed up to 23 cotton varieties that were quantified for Cry1Ac protein and insect bio-assayed for *H. armigera*. There were some varieties that were selected from more than one district and some others were selected only once. Highest average expression (2.65 µg g^−1^) in the UC was observed from variety named Jullundur seeds with 90% insect mortality. Minimum average *Cry1Ac* expression (0.26 µg g^−1^) was recorded for Z-33 with 28% insect mortality in the UC (Table S8) except NIAB-Kiran (Fig S2). Average expression in the MC for 23 varieties varied from 0 to 0.96 µg g^−1^. Highest MC expression (0.96 µg g^−1^) was recorded for BS-70, Cemb-33 and Jullundur Seeds with 67, 68, and 70% insect mortality, respectively. Lowest MC expression (0.20 µg g^−1^) was recorded for VH-179 with 13% mortality of *H. armigera* (Table S9).

No varieties, except Cemb-33 (0.13 µg g^−1^) and BS-15 (0.05 µg g^−1^) showed any expression in LC. Both these varieties recorded 10% insect mortality (Table S10). Average Cry1Ac expression in TDB varied from 0 µg g^−1^ (NAIB-Kiran) to 0.89 µg g^−1^ (BS-80) and insect mortality varied from 0 to 57% respectively. Lowest expression in TDB was observed for VH-179 (0.18 µg g-1) with 11% insect mortality (Table S11). Likewise, expression in the squares was relatively higher than MC, LC and TDB. Highest Cry1Ac expression and insect mortality was observed for Cemb-33. Lowest expression (0.24 µg g-1) for *Cry1Ac* with 15% insect mortality was observed for MNH-1020 (Table S12).

### 3.6. *Lethal dose of Cry1Ac for* effective control of H. armigera

**Figure 5.**
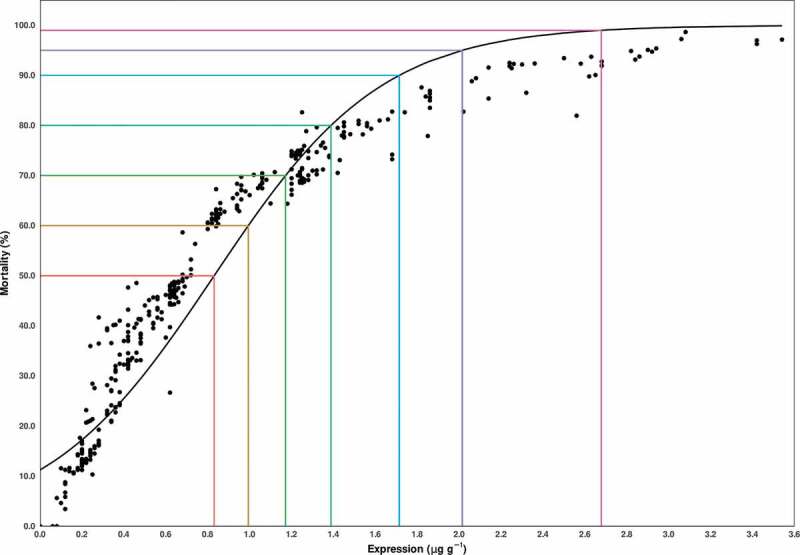
Estimate of lethal doses of Cry1Ac endotoxin. Expression denotes Cry1Ac endotoxin expression (from 0.0 µg g^−1^ to 3.6 µg g^−1^) whereas mortality describes the mortality percentage of *H. armigera* insect (from 0 to 100%). Five healthy plants were used for each bioassay study as biological replicate.

Logistic regression analysis revealed that Cry1Ac expression has a tight linkage with *H. armigera* mortality (*p* < .001). The estimated odds of *H. armigera* mortality multiply by 12.03 for each unit increase in expression; that is, there is an 1103% increase. Based on logistic regression model estimates of LD50, LD95 and LD99 are 0.84 µg g^−1^, 2.20 µg g^−1^ and 2.67 µg g^−1^ respectively (Figure 5).

### 3.7. Cry1Ac endotoxin association with farmer reported inputs (Fertilizer, irrigation and rainfall)

Linear model regression was used to study the effect of all inputs on Cry1Ac expression like; number of irrigations, rainfall and fertilizer. Three model estimates were used as shown in Table 3. If all factors, i.e.,, rainfall, irrigation and fertilizer will be considered as zero, then *Cry1Ac* expression will be 0.39 µg g^−1^ as was suggested by model 1. Number of irrigations and micronutrients showed no significant effect on *Cry1Ac* expression (Table 3). Minimum number of irrigation applied by any farmer was 5. Some farmers were applying above 20 irrigations (Table S13), so irrigations beyond 5 will not benefit in enhancing *Cry1Ac* expression.

**Table 3.** Linear regression model estimate of farmers reported inputs with Cry1Ac expression.

	Dependant variable		
Independent variables	Model 1	Model 2	Model 3
Constant	0.387**	0.396**	0.406**
Number of irrigations	0.014		
Rain Fall (low)	−0.464***	−0.400***	−0.437***
Rain fall (normal)	−0.434**	−0.439**	−0.342**
Nitrogen fertilizer	0.259***	0.278***	0.268***
Phosphorus fertilizer	0.440***	0.456***	0.518***
Potassium fertilizer	0.028**	0.026**	0.032***
Micronutrients	0.007	0.008*	
R^2^	0.737	0.730	0.718
Adjusted R^2^	0.715	0.710	0.702
Residual Standard Error	0.386 (DF = 81)	0.389 (DF = 82)	0.394 (DF = 83)
F Statistics	32.466*** (DF = 7; 81)	36.858*** (DF = 6; 82)	42.367*** (DF = 5; 83)

Rainfall, nitrogen, phosphorous and potassium fertilizer have direct effects on *Cry1Ac* expression. It was noticed that if rainfall decreases from high to normal then expression also decreases by 0.342 µg g^−1^. However, if rainfall is low *Cry1Ac* expression will be 0.44 µg g^−1^ low as compared to same situation if rainfall is normal. Likewise, addition of one bag of 50 Kg per acre of nitrogenous fertilizer will increase expression by 0.27 µg g^−1^. By keeping all other factors constant, addition of one bag of 50 Kg of phosphorus per acre, e.g.,, DAP or SSP will increase expression by 0.52 µg g^−1^. However, in case of potassium, addition of 1 Kg of potassium per acre will increase expression by 0.032 µg g^−1^ (Table 3).

## 4. Discussion

Chemical pesticides were first introduced in Pakistan during 1970s for control of cotton bollworms. Later on, farmers found themselves on a pesticide “treadmill” forcing them to use stronger doses. As a result, the frequency of toxic chemical pesticide sprays increased exponentially during 1990s^26^ and bollworms remained uncontrolled and pesticides poisoning cases were reported frequently.^27–29^ As a safe solution, Bt cotton technology was introduced by informal way in mid 2000s.^30^ Later on, many Bt cotton varieties were developed locally by using Monsanto-based *Cry1Ac* gene (Mon531 event) to harvest economic benefits^5,31^ and NBC officially approved Bt cotton cultivation in 2010. *Cry1Ac* gene alone is losing its effectiveness against *H. armigera* and farmers have started using sprays again (Fig S3). On the other hand, approval for latest Bt technologies, i.e., Bollgard II (*Cry1Ac* + *Cry2Ab* genes) and Bollgard III (*Cry1Ac* + *Cry2Ab* + *Vip3Aa* genes) with broad spectrum insecticidal activity is still pending.^32^ Following section explains the mechanisms of evolution of resistance in *H. amigera* and its possible causes and solutions.

### 4.1. Field-evolved resistance

Cultivation of transgenic cotton possessing *Cry1Ac* (Mon531 event) has diminished pesticide application against bollworms, especially *H. armigera*. Nevertheless, extensive Bt cotton cultivation resulted in field-evolved resistance in lepidopteron pests at various localities and reducing pest control and effectiveness.^9,33^ Expression and bioassay studies were conducted from UC, MC and LC, squares and TDBs of 89 Bt cotton varieties (Figures 2 and 3). The advantage of our study as compared to previous studies,^17,19^ is that leaf samples used in this study were directly collected from farmers’ field rather than growing in controlled conditions. Additionally, the number of varieties used in this study is too large in comparison to previous studies 9.^19^ and 26^17^ In addition, expression and bioassay studies were conducted at different plant parts such as TDBs and Squares that have not been covered in previous studies. Although quantification of Cry1Ac endotoxin from bolls has been done,^34^ bioassay were not conducted previously.

**Figure 6.**
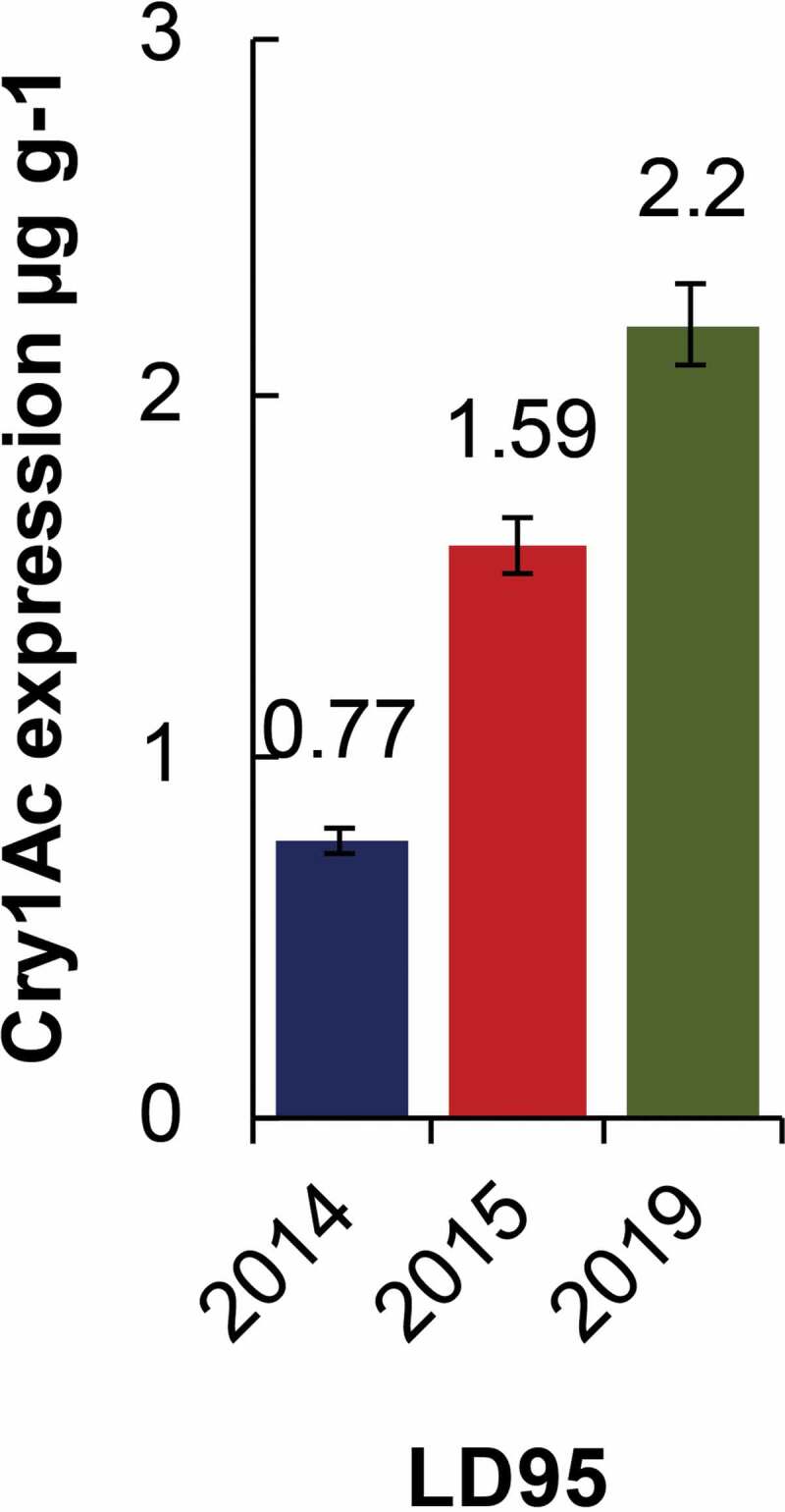
Comparison of LD95 of previous and current studies. The figure indicates that *H. armigera* is developing resistance and updated resistance from 0.77 µg g-1 to 2.20 µg g-1 from 2014–2019.

Previously the level of Cry1Ac endotoxin as low as 0.77 µg g-1 of fresh leaf weight was sufficient to kill 95% insect population of *H. armigera*.^19^ However, with the passage of time insects in Punjab are developing resistance against *Cry1Ac* (Mon531 event). This phenomenon has been studied during 2015, but published in 2019, and found that the LD95 (1.59 µg g-1) increased by two folds of the previously reported value.^17^ Interestingly, in our study the value further raised up to 2.20 µg g-1 (Figure 5). The comparison of findings of this study with reference to previous studies is summarized in Figure 6. This comparison of LD95 dictates that *H. armigera* is evolving resistance against Bt cotton possessing *Cry1Ac* (Mon531 event).

Field-evolved resistance against *Cry1Ac* by *H. armigera* was reported previously from China,^16,35-39^ India,^40^ Australia,^41^ USA^42^ and West Africa.^43^ Apart from *H. armigera* there are several other reports of resistance development such as *Pectinophora gossypiella*,^12,44^ *Helicoverpa zea, Heliothis virescens*^42^ and *Diatraea saccharalis*.^45^ Insects develop resistance due to inconsistent and variable expression of *Cry1Ac* in plant tissues resulting in gradual increase in LC50 over a period of 10–15 years in absence of proper resistance management measurements.^35^ As is the case with Pakistan, LD95 has increased approximately three folds within a period of 6 years (Figure 6) providing strong evidence of resistance development against Bt cotton. If resistance is not properly managed then it will lead to serious consequences.

### 4.2. Variable expression of Cry1Ac in different plant parts

Sustainable expression in Bt cotton possessing *Cry1Ac* is necessary for control of insects/pest especially bollworms. The results showed that Bt expression was highly variable among different canopies, i.e., UC, MC and LC and fruiting parts. Comparison of Cry1Ac endotoxin and insect mortality% from leaf bio-toxicity assay of different plant parts of IUB-13 cotton variety is presented in Fig S4. Maximum expression and insect mortality percentage is observed in UC leaves followed by MC leaves, squares, TDBs and lowest observation for both parameters in LC leaves. The possible reason behind low insect mortality from LC and MC are hardness of leaves^17^ or low Cry endotoxin expression. The leaves from MC and LC were relatively old aged and were harder hence were not preferable by *H. armigera* larvae which leads to starvation and lose weight but low mortality. However, one earlier study also reported symptoms of cannibalism in leaf bio-toxicity studies from harder parts of plant^17^ which ultimately leads to insect mortality from these parts in bioassay. Concentrations of Cry endotoxin in MC and LC and even bolls was not significant enough to kill the insect but disliking of insects toward these plant parts led to starvation and cannibalism and insect mortality. However, survival rate was high in these plant parts (Figure 2).

*H. armigera* larvae are more comfortable with UC leaves as these are succulents and at the same time has high Cry endotoxin hence high insect mortality observed on the UC leaves.^46^ Previous studies have also reported that Bt toxin vary significantly among different tissues/plant parts in cotton plant during its life-cycle.^22,47^ The variable expression of Cry protein in different plant canopies and fruiting parts provides evidences of evolution of resistance in insects. Bollworms attack on the UC however some insects are transferred to MC and LC and lead to accumulation of sub-lethal level of Cry endotoxin without its death. As a result, insects start developing resistance against Cry endotoxin and this is what happening at the farmers’ fields and claim of farmers about survival of *H. armigera* on Bt cotton varieties seems rational. These results also help in understanding the late season survival of American bollworm on Bt cotton. Further *Cry1Ac* expression declines exponentially during crop growth season and drops down lethal concentration that provides shelter to insect.^21^

### 4.3. Variable expression of Cry1Ac in different varieties

Another possible explanation of evolution of resistance is; highly variable level of Cry1Ac endotoxin (0.26–3.42 µg g^−1^) among Bollgard-I cotton cultivars grown and multiplied at farmers field all over Punjab. Out of 88 Bt cotton varieties, 61 varieties possessed Cry1Ac endotoxin below LD95 2.20 µg g^−1^ (Figure 2). Factors that contribute mainly to variable expression of Cry endotoxin among cultivars are, i.e., variation in base sequences, copy number, insertion point of gene and promotor used.^48^ However, all these factors were common because all the commercial Bt cotton cultivars grown in Punjab Pakistan contain *Cry1Ac* (Mon531 event).^6^ We speculate that high level of variability among genotypes may be either due to methylation of promotor or due to variable genetic backgrounds.^47^

About 50% farmers from low land owning reported that they use their homemade seed for 2–3 years. Whereas Cry endotoxin expression in saved seed (homemade seed) is significantly lower than the seed bought from authorized dealers from market.^49^ Other buy seed from their fellow farmers and only progressive growers visit Punjab Seed Corporation (PSC) Offices or authorized dealers and even they also do not have any mechanism to check purity and concentration of Cry endotoxin in it. Despite of the reports on LD95^17,19^ there is no standard protocol at Govt. level for approval of Bt cotton varieties and no minimum toxic limit is monitored in Bt varieties before approval.^6^ Although reports are available to control insect but these only lies with the literature with no role to play in policy making. Usually, cotton breeders keep on monitoring all other traits with special focus on yield during variety evolution process and at final stages it is sent for quantification of Bt trait which shows their seriousness about the gravity of problem. Unchecked marketing of Bt cotton seed is another leading cause of poor performance of transgenic technology and evolution of resistance.^6,50^

### 4.4. Effect of location and agronomic practices on Cry1Ac expression

Variable geographic conditions also affect *Cry1Ac* expression. IUB-13, which was grown in 14 out of 15 districts showed variable Cry endotoxin expression i.e. 0.34 to 3.42 µg g^−1^ (Figure 2) with insect mortality varying from 37 to 96%, respectively (Figure 3). These results provide strong evidence that geographical conditions affect Cry endotoxin expression as also elaborated by.^49^ Further, different inputs such as irrigations, rainfall, micronutrients, and macronutrients (Nitrogen, phosphorous, and potassium) also affect cry endotoxin expression (Table 3). Cry1Ac expression was positively regulated by nitrogen application in contrast to previous findings of.^49^ Effect of irrigation was non-significant whereas rainfall, potassium, and phosphorous showed positive association as explained already.^49^ These results have serious policy implication to restrict unplanned and unbalanced fertilizer application. Along with nitrogen phosphorus, potash and micronutrients also significantly affect cotton growth and Cry endotoxin expression.^51^ Use of balanced fertilizer is highly recommended for good endotoxin expression and crop stand.

### 4.5. Trait purity and introduction of new Bt and RR technology

Adaptation rate of Bt cotton was nearly 100%. Yet there appears certain disconnect between what farmers believe they are cultivating and what they are actually cultivating. 10% samples were false negative whereas 27% were false positive (Table 1). Spielman also reported similar results about deviation in farmers claim about planting of Bt cotton.^6,10^ Cross pollination and seed mixing during production and supply chains also decrease expression of Cry endotoxin and favors development of resistance in *H. armigera*.^17^

Cultivation of new Bt genes, i.e., *Cry2Ab* and *Vip3Aa* was negligible. Only 0.5% samples were found positive for *Cry2Ab* and not a single sample contained *Vip3Aa* gene. Both of these genes have different receptor sites and have role in delaying insect resistance in particular.^9,52^ Use of more than Cry genes in cotton plants is used as a delaying tactics for controlling insect resistance. Cry genes diversity needs to be maintained in the cotton field under a timeframe for sustainable resistance and enhancement of shelf life of Bt cotton varieties. Repeated use of single Bt gene (*Cry1A*c, event MON531) is resulting in development of insect resistance.^53^ Similarly, 2.2% samples contained glyphosate-tolerant (*CP4, EPSPS*) gene (Table 2). *EPSPS* gene enables crops to withstand post emergent glyphosate herbicide application for complete weed control.^54^ Reasons for low spread of latest technologies is unavailability of agreement with Monsanto for purchase of rights.^10^ Farmers are getting seed by black marketing and mostly, dealers are selling the false seed.

### 4.6. Suggestions for breeders, policy makers and farmers

The study has summarized following reasons for evolution of resistance in *H. armigera*; 1) sub-lethal level of Cry endotoxin in approved Bt cotton varieties, 2) repeated use of Bt cotton housing single Bt gene *Cry1Ac*, 3) cultivation of unapproved varieties, 4) variable expression of Cry endotoxin in different plant parts, 5) unbalanced use of fertilizer, and 6) outdated agronomic practices. There is a need of serious policy interventions to address these issues. Regulatory bodies should conduct bioassay studies for *H. armigera* against *Cry1Ac* at defined intervals not exceeding 3 years to revise lethal dose standards. Because our results and findings of^17^, depict that LD95 for *H. Armigera* is increasing at rapid pace. Minimum Cry endotoxin level should be strictly monitored before approval of any candidate variety. Cotton breeders should check Bt/non-Bt status and Cry endotoxin level of promising plants in F_2_ and successive generations.^6^

Highly variable expression in different plant parts needs to be addressed through consistent gene expression.^55^ Use of Cry2Ah1 gene has ability to develop broad-spectrum resistance against cotton bollworms^56^ and may helpful in delaying insect resistance. Mixture of Bt with non-Bt cotton seeds may be used as an option to delay insect resistance. However, some author states that it will improve evolution and dominance nature of resistance.^9,57^ Most widely used strategy for delaying insect resistant is growing of refugee crop. Resistance insects on Bt plants may mate with relatively abundant susceptible insects on refugee crop. If resistance is controlled by recessive alleles the resulting heterozygous insect will die on Bt plants.^9,11^ Production technology of cotton crop needs to be revisited as recommendations for Bt cotton cultivation are not updated according to changing climate.^58^ Leading Government Agricultural Institute should devise new production technology on scientific grounds which should be shared with farmers through extension services.

## 5. Conclusion

Accumulation of Cry endotoxin in Bt cotton is not a simple phenomenon and it is highly influenced by genetics, sampling time during growing season, geography, climatic conditions and agronomic practices. Bt cotton expression was found highly variable among different plant parts, i.e.,highest Cry endotoxin accumulation and mortality of *H. armigera* was observed on upper canopy and lowest expression and insect control was found from the lower canopy. Similarly, all Bt accessions also varied significantly for Cry endotoxin expression and insect mortality. Our results suggested that Bt varieties expressing Cry endotoxin below 2.20 µg g-1 may not be registered/approved as Bt varieties. Bt cotton varieties cultivated by farmers were found highly impure by mixing of on-Bt seed. Different reasons for the evolution of practical resistance in *H. armigera* against *Cry1Ac* were recorded as; 1) sub-lethal level of Cry endotoxin in Bt varieties, 2) cultivation of unapproved varieties, 3) variable expression of Cry endotoxin across different canopies and various fruiting parts, 4) unbalanced use of fertilizer and 5) poor agronomic management practices. To address these issues a multidisciplinary approach was proposed including cotton scientists and policy makers for Bt cotton revival.

## Supplementary Material

Supplemental Material
