# Supplementary material for: The level of Cry1Ac endotoxin and its efficacy against H. armigera in Bt cotton at large scale in Pakistan
Source: GM Crops Food. 2020 Aug 6;12(1):1–17. doi: 10.1080/21645698.2020.1799644 (PMC7553749; doi:10.1080/21645698.2020.1799644)
Supplement: Supplemental Material [file KGMC_A_1799644_SM5272.docx]

Supplementary Material

# Supplementary Materials and Methods

## ELISA Procedure:

After thorough mixing of leaf extract with 1X extraction buffer, samples were centrifuged at 5000 rpm for 05 minutes and supernatant was collected in separate eppendorf tubes. Wash buffer was prepared from wash buffer salt provided in commercial quantification kit (QuantiPlateTM Kit, EnviroLogix, Inc., USA). Supernatant extract was diluted as described in the manual supplied with kit. For 1:11 dilution, 1X extraction buffer @ 500 µl and 50 µl samples extract was added to dilution tube labelled for each sample and mixed thoroughly.

After sample preparation 100 µl of negative control, 100 µl of each calibrator (2, 4, 8 and 16 ppb), and 100 µl of each sample extract was added to their respective wells according to layouts. Contents of the wells were thoroughly mixed by placing plate in strip holder and moving in circular motion for 20-30 seconds. Wells were covered with tape or Parafilm to prevent evaporation and incubated at ambient temperature for 15 minutes. 100 µl of Cry1Ab-Enzyme conjugate buffer was added to each well and contents of the wells were mixed thoroughly however intensive care was taken to avoid cross contamination at each step. Covered the wells with tape or Parafilm again and incubated at ambient temperature for 01 hour and shake at 200 rpm. After incubation, contents of the wells were shaken rigorously into the sink. Flooded wells with wash buffer and empty it and repeated this step thrice. Slapped inverted plate several times on a paper towel and removed maximum possible water and added 100 µl of substrate. Covered plate with tape or Parafilm and incubated again for 30 minutes at ambient temperature and placed in an incubator shaker at 200 rpm. At final step added 100 µl of stop solution which turned well contents to yellow colour. Plate was then placed in µquant Bio Tek micro plate reader and GEN5 software was used for curve analysis. Final readings were recorded in parts per million (ppm) which corresponds to microgram per gram (µg/g).

## Detail procedure for bioassay of ten days bolls and Squares:

For that 09 squares from each variety were collected and petiole of each square was wrapped in wet tissue and placed in glass petri plates (6” diameter) (03 squares in one petri plate) supplied with five 2nd instar larvae. For bioassay of bolls 06 bolls of approximate 10 days age were collected from each variety and their exocarp was removed and place in glass petri plate (6” diameter) 2 bolls in one petri plate.

# Supplementary Figures and Tables

## Supplementary Figures


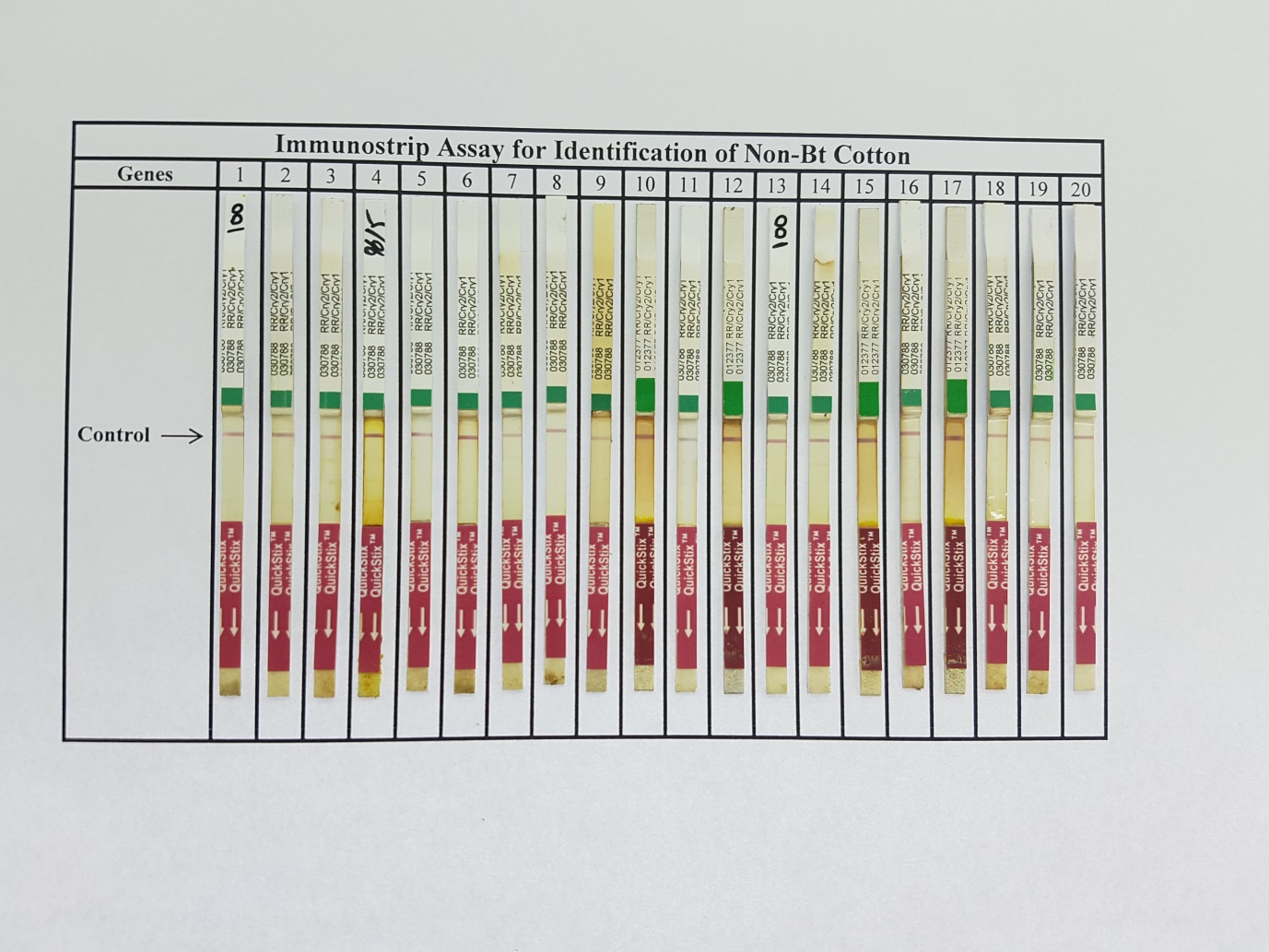


**Fig S1a. Results of strip test of 20 cotton samples for identification of Non-Bt cotton.**


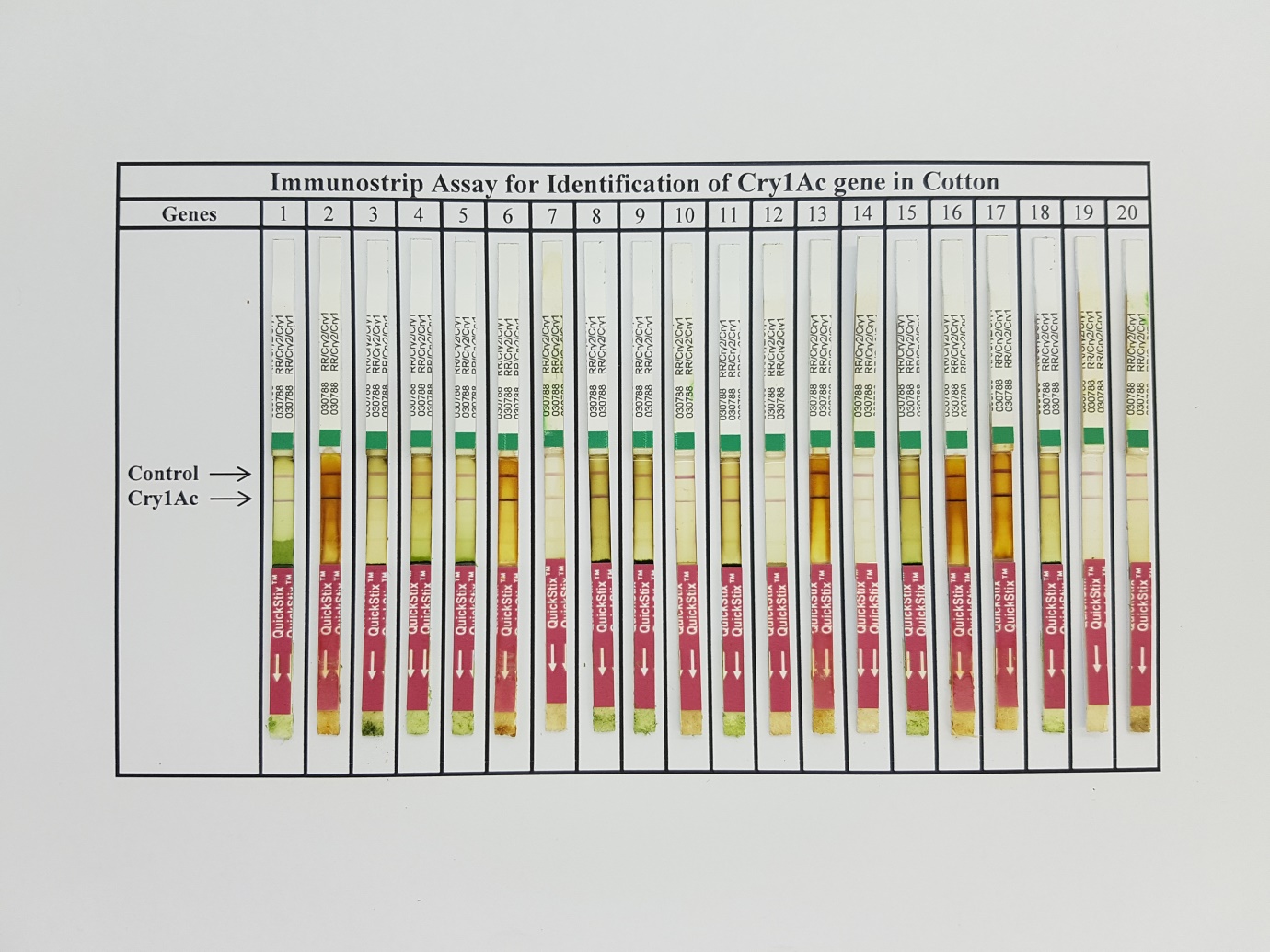


**Fig S1b. Results of strip test of 20 cotton samples for identification of Bt cotton possessing Cry1Ac gene.**


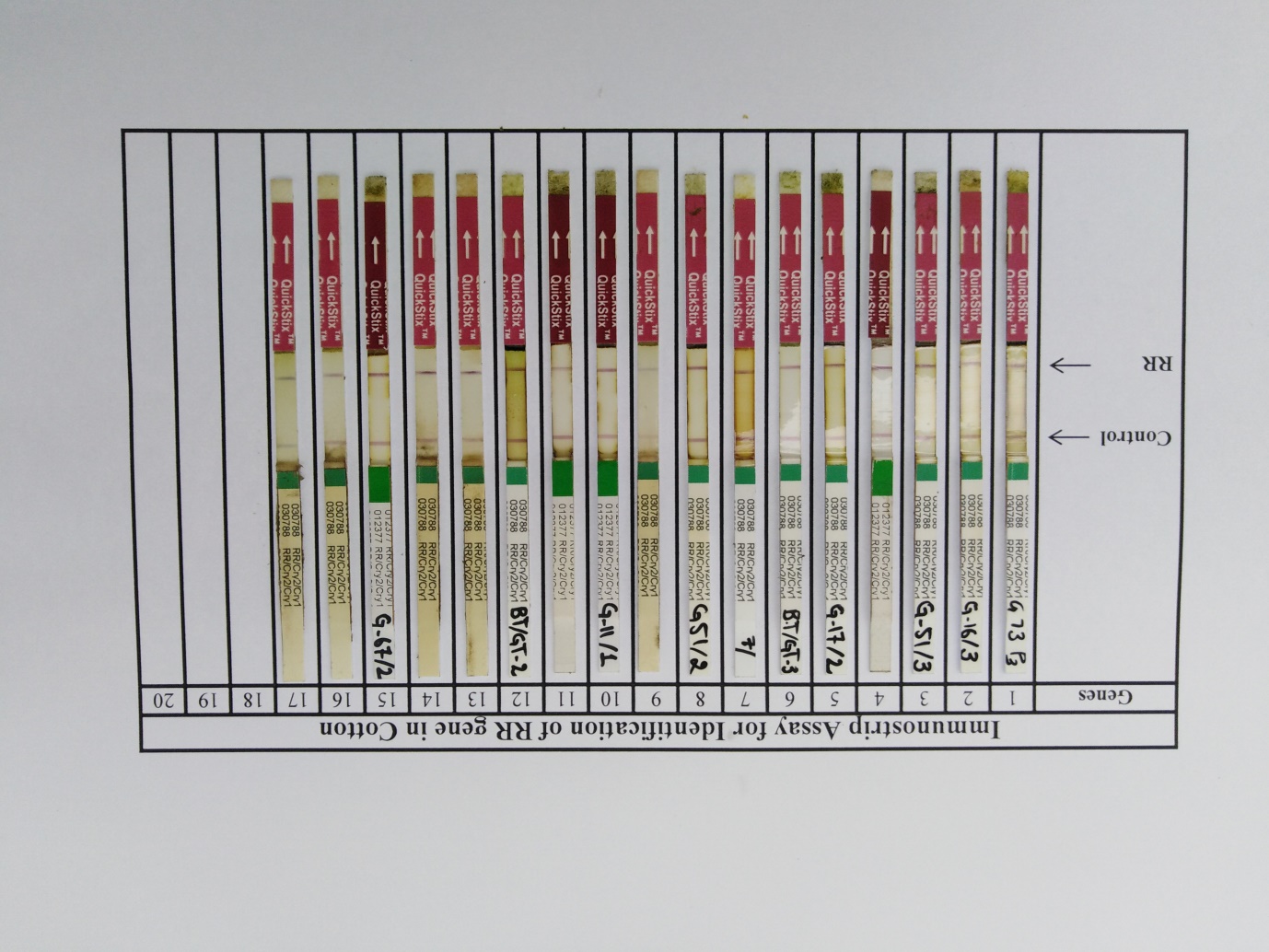


**Fig S1c. Results of strip test of 20 cotton samples for identification of RR gene.**


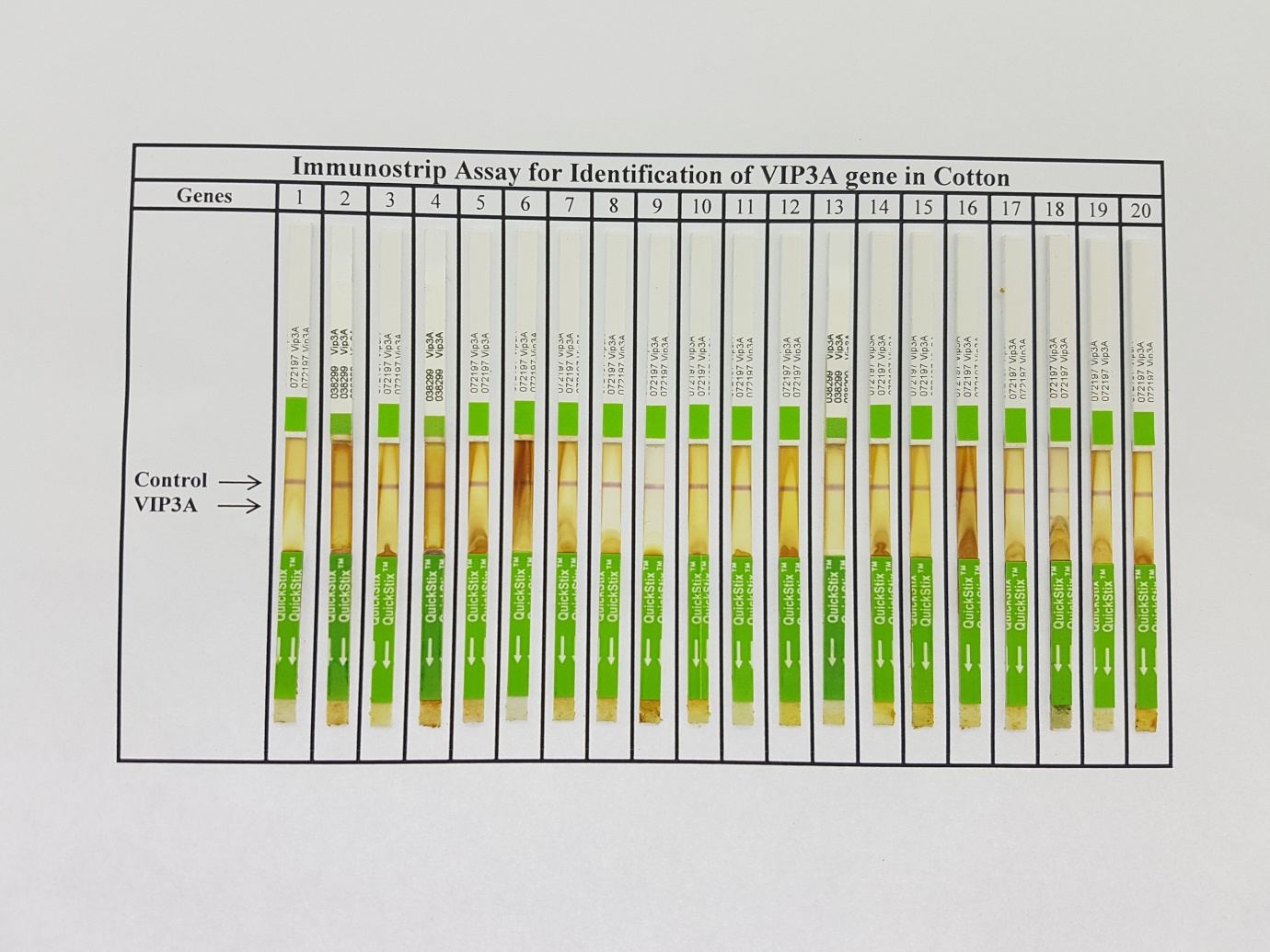


**Fig S1d. Results of strip test of 20 cotton samples for identification of Bt cotton possessing Vip3Aa gene.**


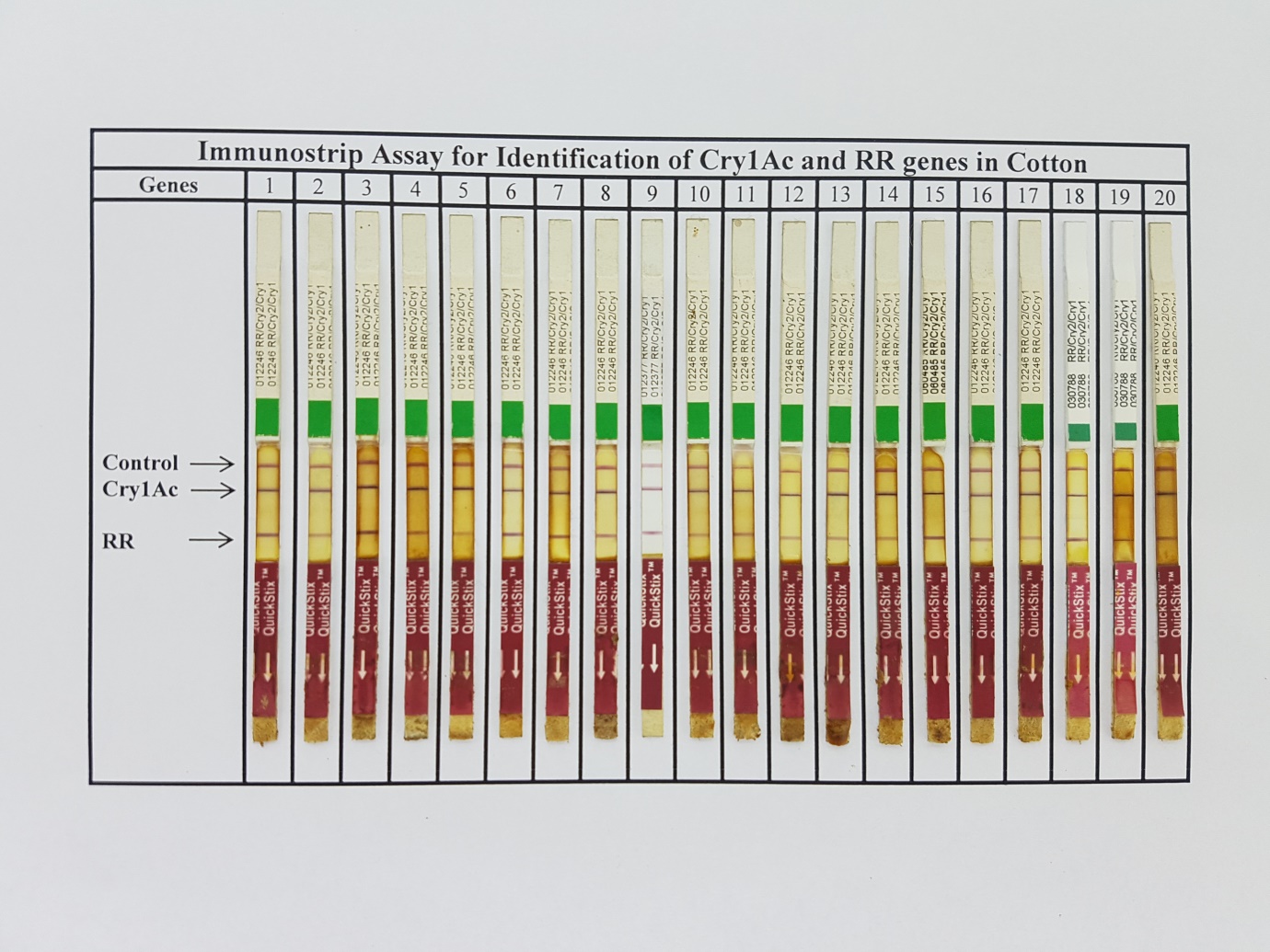


**Fig S1e. Results of strip test of 20 cotton samples for identification of Bt cotton possessing Cry1Ac and RR genes.**


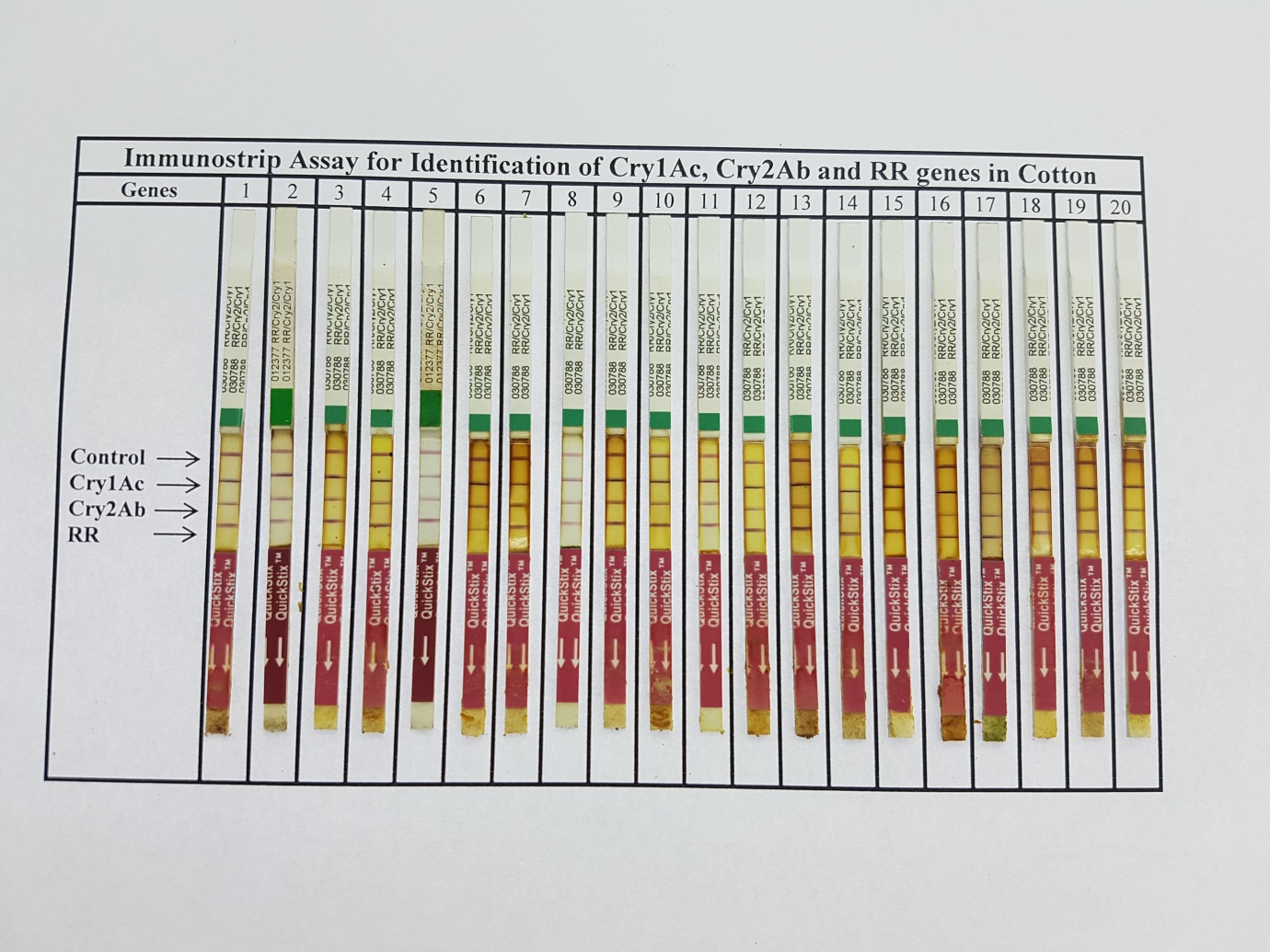


**Fig S1f. Results of strip test of 20 cotton samples for identification of Bt cotton possessing Cry1Ac, Cry2Ab and RR genes.**

##
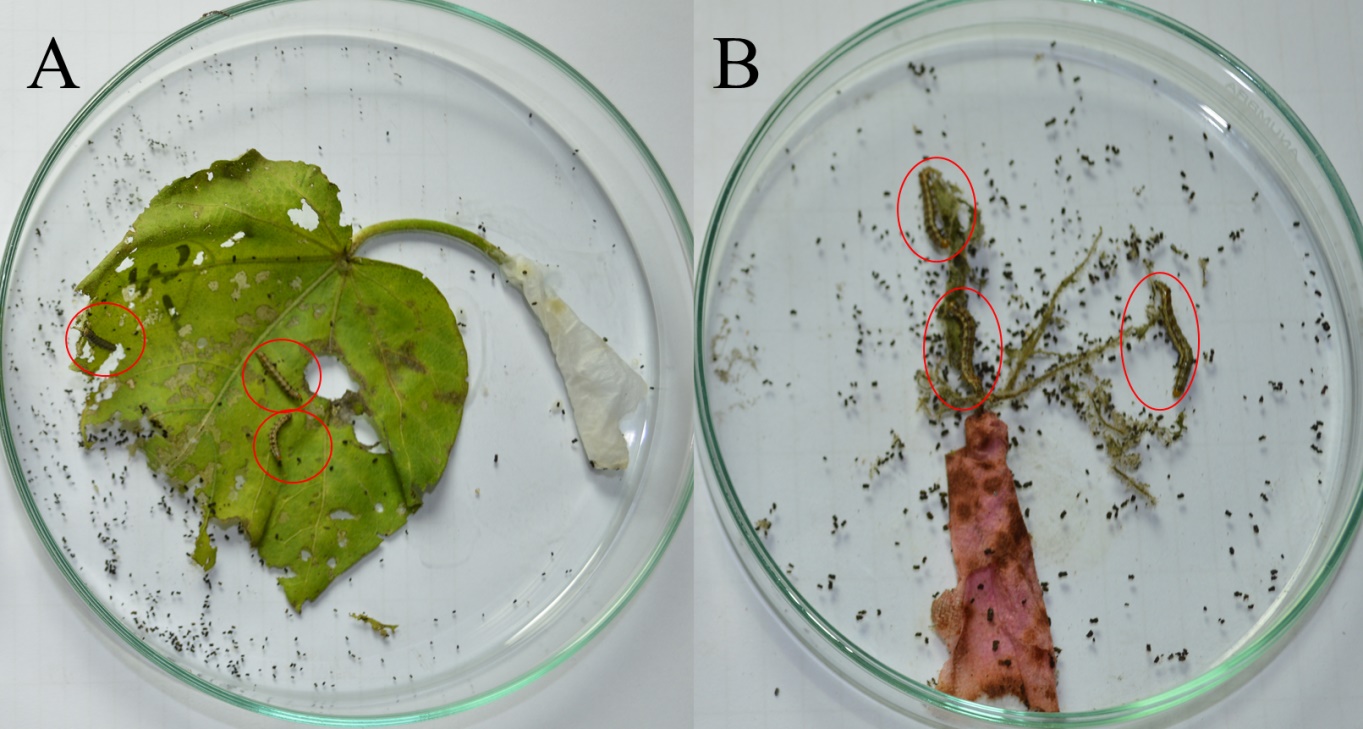


**Fig S2. Leaf bio-toxicity assay of Bt and Non-Bt cotton varieties using 2nd instar H. armigera larvae. A: Dead larvae of H. armigera after eating toxic leaf of Bt cotton. B: Alive larvae of H. armigera nourishing on leafs of Non-Bt cotton.**


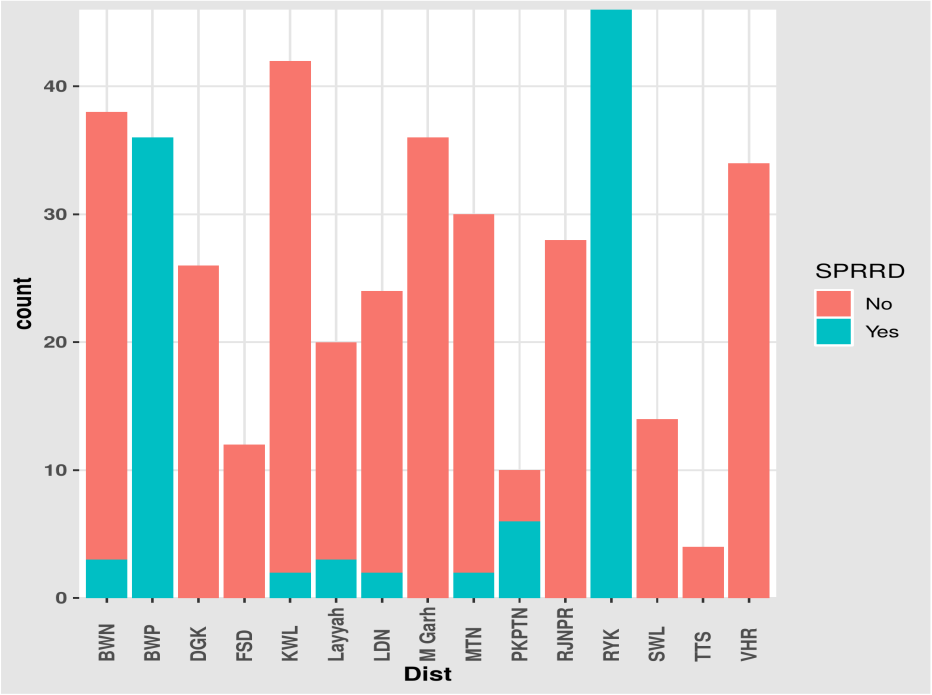


**Fig S3. Opinion of farmers about Bt cotton effectiveness and reduction in number of sprays by Bt cotton. Whereas SPRRD describes number of spray reduced or not and Yes and NO indicates farmers opinion. BWP (Bahawalpur), BWN (Bahawalnagar), RYK (Rahim Yar Khan), DGK (Dera Ghazi Khan), M Garh (Muzaffargarh), RJNPR (Rajanpur), MTN (Multan), LDN (Lodhran), KWL (Khanewal), VHR (Vehari), SWL (Sahiwal), PKPTN (Pakpattan), FSD (Faisalabad), TTS (Toba Tek Singh).**


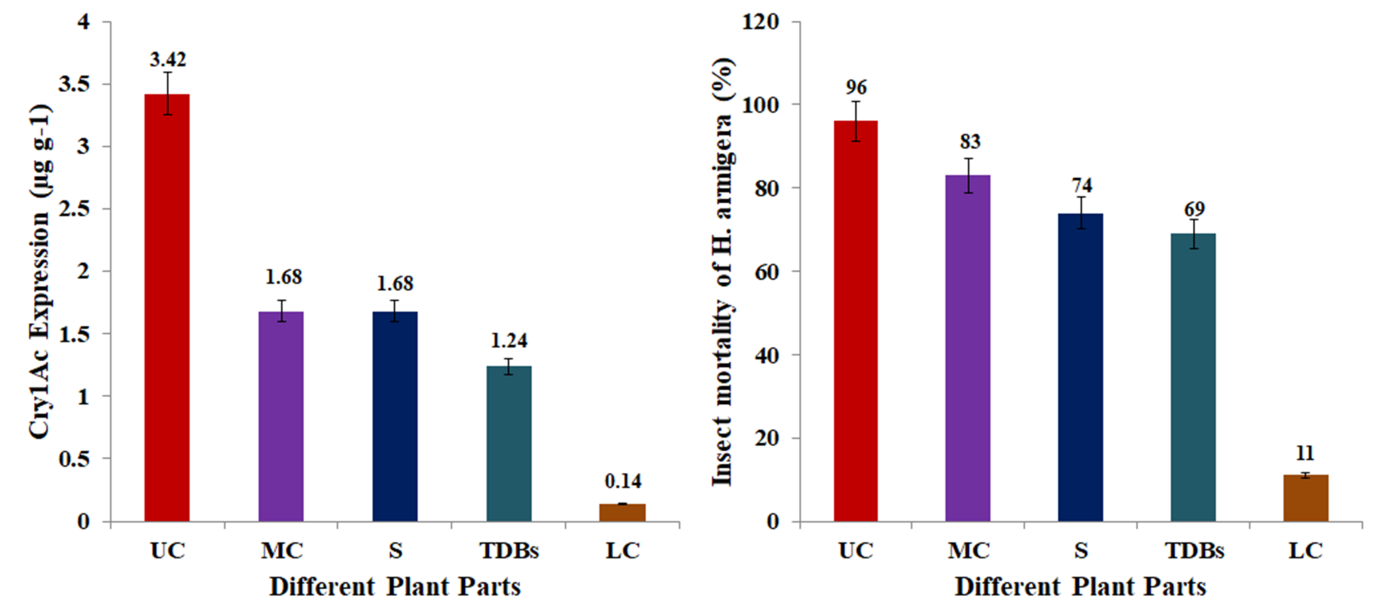


**Fig S4. Comparison of Cry1Ac expression and mortality of H. amrigera in leaf bio-toxicity assay from different plant parts of IUB-13 cotton variety. Whereas Upper Canopy abbreviated as (UC), Middle Canopy (MC), Lower Canopy (LC), Squares (S) and Ten Days Bolls (TDBs).**

**Table S1. Detail of 400 farmers surveyed from 5 Divisions, 15 Districts and 47 Tehsils of Punjab with cotton cultivation area for year 2018 as per Director General Agriculture Extension Punjab.**

| Sr. # | | Divisions of Punjab | | Districts of Punjab | | Tehsils of Punjab | Locations Surveyed |
| --- | --- | --- | --- | --- | --- | --- | --- |
| 1 | | Multan  Area: 1959.66 (000acres) | | 1. [MULTAN](http://www.politicpk.com/multan-district-uc-list-mna-mpa-seats-detail-%d9%85%d9%84%d8%aa%d8%a7%d9%86-%d8%b6%d9%84%d8%b9-%da%a9%db%8c-%db%8c%d9%88%d9%86%db%8c%d9%86-%da%a9%d9%88%d9%86%d8%b3%d9%84%d8%b2/)   Area: 437.08 (000acres) | | 1. JALALPUR PIRWALA 2. MULTAN CITY 3. MULTAN SADDAR 4. SHUJABAD | 30 |
|  |  |  |  | 1. [VEHARI](http://www.politicpk.com/vehari-district-uc-list-mna-mpa-seats-%d9%88%db%81%d8%a7%da%91%db%8c-%d8%b6%d9%84%d8%b9-%da%a9%db%8c-%db%8c%d9%88%d9%86%db%8c%d9%86-%da%a9%d9%88%d9%86%d8%b3%d9%84%d8%b2/)   Area: 533.71 (000acres) | | 1. BUREWALA 2. MAILSI 3. VEHARI | 34 |
|  |  |  |  | 1. [LODHRAN](http://www.politicpk.com/lodhran-district-uc-list-mna-mpa-seats-%d9%84%d9%88%d8%af%da%be%d8%b1%d8%a7%da%ba-%d8%b6%d9%84%d8%b9-%da%a9%db%8c-%db%8c%d9%88%d9%86%db%8c%d9%86-%da%a9%d9%88%d9%86%d8%b3%d9%84%d8%b2/)   Area: 490.00 (000acres) | | 1. DUNYAPUR 2. KAHROR PACCA 3. LODHRAN | 24 |
|  |  |  |  | 1. [KHANEWAL](http://www.politicpk.com/khanewal-district-uc-list-%d8%ae%d8%a7%d9%86%db%8c%d9%88%d8%a7%d9%84-%d8%b6%d9%84%d8%b9-%da%a9%db%8c-%db%8c%d9%88%d9%86%db%8c%d9%86-%da%a9%d9%88%d9%86%d8%b3%d9%84%d8%b2/)   Area: 498.88 (000acres) | | 1. JEHANIAN 2. KABIRWALA 3. KHANEWAL 4. MIAN CHANNU | 42 |
| - Total Number of Farmers in Multan Division | | | | | | | - 130 |
| 2 | | DERA GHAZI KHAN  Area: 1149.44 (000acres) | | 1. [D.](http://www.politicpk.com/dg-khan-district-uc-list/) G. Khan   Area: 250.56 (000acres) | | 1. D.G.KHAN 2. TAUNSA 3. KOT CHUTTA | 26 |
|  |  |  |  | 1. [RAJANPUR](http://www.politicpk.com/rajanpur-district-uc-list-mna-mpa-seats-%d8%b1%d8%a7%d8%ac%d9%86-%d9%be%d9%88%d8%b1-%d8%b6%d9%84%d8%b9-%da%a9%db%8c-%db%8c%d9%88%d9%86%db%8c%d9%86-%da%a9%d9%88%d9%86%d8%b3%d9%84%d8%b2/)   Area: 373.03 (000acres) | | 1. JAMPUR 2. RAJANPUR 3. ROJHAN | 28 |
|  |  |  |  | 1. [LAYYAH](http://www.politicpk.com/layyah-district-uc-list-mna-mpa-seats-%d9%84%db%8c%db%81-%d8%b6%d9%84%d8%b9-%da%a9%db%8c-%db%8c%d9%88%d9%86%db%8c%d9%86-%da%a9%d9%88%d9%86%d8%b3%d9%84%d8%b2/)   Area: 137.08 (000acres) | | 1. CHAUBARA 2. KAROR LAL ESAN 3. LAYYAH | 20 |
|  |  |  |  | 1. [MUZAFFARGARH](http://www.politicpk.com/muzaffargarh-district-uc-list-mna-mpa-seats-detail-%d9%85%d8%b8%d9%81%d8%b1%da%af%da%91%da%be-%d8%b6%d9%84%d8%b9-%da%a9%db%8c-%db%8c%d9%88%d9%86%db%8c%d9%86-%da%a9%d9%88%d9%86%d8%b3%d9%84%d8%b2/)   Area: 388.76 (000acres) | | 1. ALIPUR 2. JATOI 3. KOT ADDU 4. MUZAFFARGARH | 36 |
| - Total Number of Farmers in DG Khan Division | | | | | | | 110 |
| 3 | BAHAWALPUR  Area: 1847.19 (000acres) | | 1. [BAHAWALPUR](http://www.politicpk.com/bahawalpur-district-uc-list-mna-mpa-seats/)   Area: 742.70 (000acres) | | 1. AHMADPUR EAST 2. BAHAWALPUR CITY 3. BAHAWALPUR SADDAR 4. HASILPUR 5. KHAIRPUR TAMEWALI 6. YAZMAN | | 36 |
|  |  |  | 1. [BAHAWALNAGAR](http://www.politicpk.com/bahawalnagar-district-uc-list-mna-mpa/)   Area: 667.42 (000acres) | | 1. BAHAWALNAGAR 2. CHISHTIAN 3. FORT ABBAS 4. HAROONABAD 5. MINCHINABAD | | 38 |
|  |  |  | 1. [RAHIM YAR KHAN](http://www.politicpk.com/rahim-yar-khan-district-uc-list-mna-mpa-seats-%d8%b1%d8%ad%db%8c%d9%85-%db%8c%d8%a7%d8%b1-%d8%ae%d8%a7%d9%86-%d8%b6%d9%84%d8%b9-%da%a9%db%8c-%db%8c%d9%88%d9%86%db%8c%d9%86-%da%a9%d9%88%d9%86%d8%b3/)   Area: 437.08 (000acres) | | 1. KHANPUR 2. LIAQUATPUR 3. RAHIM YAR KHAN 4. SADIQABAD | | 46 |
| - Total Number of Farmers in Bahawalpur Division | | | | | | | 120 |
| 4 | FAISALABAD  Area: 220.07 (000acres) | | 1. [FAISALABAD](http://www.politicpk.com/faisalabad-district-council-union-councils-uc/)   Area: 68.54 (000acres) | | 1. TANDLIANWALA | | 12 |
|  |  |  | 1. [TOBA TEK SINGH](http://www.politicpk.com/toba-tek-singh-district-uc-list-mna-mpa-seats-%d9%b9%d9%88%d8%a8%db%81-%d9%b9%db%8c%da%a9-%d8%b3%d9%86%da%af%da%be-%d8%b6%d9%84%d8%b9-%da%a9%db%8c-%db%8c%d9%88%d9%86%db%8c%d9%86-%da%a9%d9%88%d9%86/)   Area: 86.52 (000acres) | | 1. PIR MAHAL | | 04 |
| - Total Number of Farmers in Faisalabad Division | | | | | | | 16 |
| 5 | SAHIWAL  Area: 298.01 (000acres) | | 1. [SAHIWAL](http://www.politicpk.com/sahiwal-district-profile-uc-list-mna-mpa-seats-%d8%b3%d8%a7%db%81%db%8c%d9%88%d8%a7%d9%84-%d8%b6%d9%84%d8%b9-%da%a9%db%8c-%db%8c%d9%88%d9%86%db%8c%d9%86-%da%a9%d9%88%d9%86%d8%b3%d9%84%d8%b2/)   Area: 189.24 (000acres) | | 1. CHICHAWATNI | | 14 |
|  |  |  | 1. [PAKPATTAN](http://www.politicpk.com/pakpattan-district-uc-list-mna-mpa-seats-%d9%be%d8%a7%da%a9%d9%be%d8%aa%d9%86-%d8%b6%d9%84%d8%b9-%da%a9%db%8c-%db%8c%d9%88%d9%86%db%8c%d9%86-%da%a9%d9%88%d9%86%d8%b3%d9%84%d8%b2/)   Area: 75.65 (000acres) | | 1. ARIFWALA 2. PAKPATTAN | | 10 |
| - Total Number of Farmers in Sahiwal Division | | | | | | | 24 |
| - Total Number of Farmers Covered in Punjab | | | | | | | 400 |

**Table S2. Latitude and Longitude Observations of 400 farmers studies in Bt. Cotton Survey**

| Sr. No | Farmer Name | Lat. | Long. | Sr. No | Farmer Name | Lat. | Long. |
| --- | --- | --- | --- | --- | --- | --- | --- |
| 1 | Faiz ur Rasool | 29.575 | 72.225 | 51 | Abdul Rauf | 28.084 | 70.026 |
| 2 | Muhammad Arshad | 29.632 | 72.304 | 52 | Tariq Mehmood | 28.076 | 70.130 |
| 3 | Aqeel ur Rahman | 29.622 | 72.324 | 53 | M. Arshad | 28.487 | 70.618 |
| 4 | Mian Asad Abbas | 29.595 | 72.295 | 54 | Zohaib Arshad | 28.526 | 70.563 |
| 5 | Abdul Rasheed | 29.695 | 72.558 | 55 | M. Rafique | 28.619 | 70.653 |
| 6 | Baber Hussain | 29.670 | 72.577 | 56 | Ch. Riaz Ahmed | 28.667 | 70.726 |
| 7 | Asghar Ali | 29.690 | 72.580 | 57 | M. Arshad Javed | 28.814 | 70.677 |
| 8 | Muhammad Arshad | 29.700 | 72.588 | 58 | Muhammad Nazir | 28.763 | 70.743 |
| 9 | M. Shafih Khan | 29.095 | 71.020 | 59 | Asad al Dirani | 28.854 | 70.546 |
| 10 | M. Faisal Khan | 29.105 | 71.100 | 60 | Jan Hmmad ullah | 28.753 | 70.572 |
| 11 | M. Wazeer | 29.237 | 71.068 | 61 | Awias Majeed | 28.323 | 70.336 |
| 12 | M. Khaleel | 29.346 | 71.020 | 62 | M. Khalid | 28.326 | 70.257 |
| 13 | Ghulam Mustafa | 29.146 | 71.252 | 63 | Malik Shabbir Ahmed | 28.365 | 70.423 |
| 14 | M. Imran | 29.166 | 71.262 | 64 | Waqas Ahmed | 28.333 | 70.495 |
| 15 | Ghulam Yasmeen | 29.200 | 71.287 | 65 | Muhammad Ramzan | 28.333 | 70.335 |
| 16 | Ch. Sarfraz | 29.134 | 71.265 | 66 | Malik Fahem Ahmed | 28.322 | 70.331 |
| 17 | M. Ajmal | 29.274 | 71.221 | 67 | Muhammad Nawaz | 28.456 | 70.412 |
| 18 | Riaz Hussain | 29.667 | 71.329 | 68 | Shah Nawaz | 28.499 | 70.416 |
| 19 | Allah Dita Dogar | 29.221 | 71.431 | 69 | Syed Aftab Shah | 28.533 | 70.483 |
| 20 | Abdul Rasheed | 29.236 | 71.414 | 70 | Muhammad Saleem | 28.547 | 70.474 |
| 21 | M. Nadeem | 29.194 | 71.377 | 71 | Mian Aamir | 28.548 | 70.247 |
| 22 | M. Jamil | 29.206 | 71.353 | 72 | Khalid Javed | 28.472 | 70.286 |
| 23 | Mian Abdul Ghafoor | 29.415 | 71.657 | 73 | Muhammad Asif | 28.507 | 70.261 |
| 24 | Amir Ghaffar | 29.355 | 71.644 | 74 | Muhammad Afzal | 28.601 | 70.179 |
| 25 | Asif Ali | 29.378 | 71.655 | 75 | Ch. Shamas ud Din | 28.963 | 70.738 |
| 26 | Abid Ali | 29.356 | 71.614 | 76 | Jam Muhammad Aslam | 28.890 | 70.680 |
| 27 | Rana M. Zeeshan | 29.383 | 71.871 | 77 | Umair Ali | 28.953 | 70.883 |
| 28 | Rana Liaquat | 29.415 | 71.850 | 78 | Muhammad Abdullah | 29.000 | 70.836 |
| 29 | Ishtiaq Ahmaed | 29.142 | 71.753 | 79 | Raja M. Arshad | 28.768 | 70.821 |
| 30 | Hafeez ullah | 29.140 | 71.750 | 80 | M. Asim Iqbal | 28.742 | 70.855 |
| 31 | M. Arif | 29.140 | 71.705 | 81 | Abdul Jabbar | 28.901 | 70.919 |
| 32 | Kaleem ullah | 29.149 | 71.677 | 82 | Muhammad Aslam | 28.959 | 70.956 |
| 33 | M. Tasawar | 29.133 | 71.541 | 83 | Manzoor Ahmed | 29.964 | 73.252 |
| 34 | M. Shahbaz | 29.179 | 71.611 | 84 | M. Yaseen | 29.954 | 73.232 |
| 35 | Ghulam Hussain | 29.106 | 71.498 | 85 | Mian Waris | 29.862 | 73.252 |
| 36 | Shahzad | 29.110 | 71.501 | 86 | Fazal ur Rahman | 29.947 | 73.252 |
| 37 | Munir Ahmed | 28.189 | 70.076 | 87 | M. Mushtaq | 29.811 | 73.262 |
| 38 | Moin-ud-din | 28.199 | 70.069 | 88 | M. Farooq | 29.811 | 73.254 |
| 39 | Ghulam Mustafa | 28.178 | 70.054 | 89 | Basharat Ali | 29.789 | 73.248 |
| 40 | Ghulam Mustafa | 28.316 | 70.051 | 90 | M. Sajjad | 29.789 | 73.221 |
| 41 | Liaquat Ali | 28.332 | 70.116 | 91 | Naddem Iqbal | 29.836 | 73.092 |
| 42 | Muhammad Tariq | 28.363 | 69.999 | 92 | M. Ahmad | 29.846 | 73.091 |
| 43 | Muhammad Riaz | 28.223 | 70.121 | 93 | Abdul Sattar | 29.845 | 73.037 |
| 44 | Muhammmad Hafiz | 28.188 | 70.167 | 94 | Niaz Ahmed | 29.855 | 73.082 |
| 45 | Shafique Ahmed | 28.330 | 69.852 | 95 | Farhan Ali | 30.135 | 73.600 |
| 46 | Shoukat Ali | 28.389 | 69.932 | 96 | Fakhar Hayat | 30.140 | 73.595 |
| 47 | Ghulam Moi ud din | 28.419 | 69.872 | 97 | M. Azam | 30.131 | 73.600 |
| 48 | Liaquat Ali | 28.379 | 69.880 | 98 | M. Hussain Raza | 30.190 | 73.563 |
| 49 | Faisal Kaleem | 28.214 | 69.986 | 99 | Asghar Ali | 29.629 | 73.172 |
| 50 | Khursheed Alam | 29.070 | 70.102 | 100 | Shahbaz | 29.618 | 73.143 |
| 101 | M. Nadeem | 29.637 | 73.149 | 151 | Haji Dilawar | 29.670 | 70.600 |
| 102 | M. Khadim | 29.628 | 73.144 | 152 | Zahid Khan | 29.606 | 70.573 |
| 103 | Asif Munir | 29.628 | 73.144 | 153 | Ahmed Hassan | 29.583 | 70.557 |
| 104 | Afzal Ahmed | 29.485 | 73.060 | 154 | M. Imran | 29.566 | 70.539 |
| 105 | M. Saleem | 29.219 | 72.903 | 155 | Abdul Kareem | 29.550 | 70.541 |
| 106 | M. Aslam Kamoke | 29.390 | 72.993 | 156 | Rana M. Yaseen | 29.355 | 70.468 |
| 107 | M. Ashraf | 29.390 | 72.993 | 157 | Mahar Rizwan Ahmed | 29.335 | 70.463 |
| 108 | M. Aftab | 29.220 | 72.834 | 158 | M. Qasim | 29.315 | 70.491 |
| 109 | Nasir Ahmed | 29.220 | 72.807 | 159 | M. Ali | 29.335 | 70.463 |
| 110 | M. Arshad | 29.221 | 72.786 | 160 | Jamil Buzdar | 29.315 | 70.491 |
| 111 | M. Ramzan | 29.948 | 73.345 | 161 | M. Shafih | 29.117 | 70.320 |
| 112 | Bashir Ahmed | 29.958 | 73.327 | 162 | M. Shahzad | 29.146 | 70.363 |
| 113 | Rana Iftikhar | 29.743 | 72.904 | 163 | Ijaz Ahmed | 29.144 | 70.375 |
| 114 | Rana Shakeel Ahmed | 29.746 | 72.901 | 164 | M. Arshad | 29.146 | 70.363 |
| 115 | Saddiq | 29.806 | 72.843 | 165 | M. Shafiq | 29.090 | 70.452 |
| 116 | M. Ishfaq Khan | 29.806 | 72.794 | 166 | Tahir Sajjad | 29.092 | 70.461 |
| 117 | Dilshad Mehmood | 29.798 | 72.862 | 167 | Mazhar Hussain | 29.096 | 70.48 |
| 118 | M. Aslam Javed | 29.631 | 72.841 | 168 | M.Abu Bakar | 29.095 | 70.49 |
| 110 | Ishfaq Ahmed | 29.640 | 72.841 | 169 | Saleh Muhammmad | 28.8 | 70.05 |
| 120 | M. Sarwar | 29.657 | 72.841 | 170 | Mitha Khan | 28.75 | 69.96 |
| 121 | M. Nawaz | 29.974 | 70.647 | 171 | Ali Murad | 28.72 | 69.9 |
| 122 | Ghulam Fareed | 29.971 | 70.641 | 172 | Muhammad Murad | 28.76 | 69.95 |
| 123 | Abdul Rasheed | 29.971 | 70.641 | 173 | Master Hazor Baksh | 28.71 | 69.9 |
| 124 | Ashiq Hussain | 29.931 | 70.659 | 174 | Abdul Kareem | 28.72 | 69.91 |
| 125 | Mahar M. Ayub | 29.921 | 70.694 | 175 | M. Arshad | 30.076 | 71.050 |
| 126 | M. Habib Ullah | 29.914 | 70.671 | 176 | Riaz Hussain | 30.071 | 71.034 |
| 127 | M. Ajmal | 29.914 | 70.671 | 177 | Abid Hussain Shah | 30.077 | 70.998 |
| 128 | Manzoor Ahmed | 29.879 | 70.637 | 178 | Sana Ullah | 29.874 | 70.943 |
| 129 | Ghulam Rasool | 29.839 | 70.447 | 179 | Abdul Sattar | 29.871 | 71.086 |
| 130 | M. Saeed | 29.839 | 70.448 | 180 | M. Iqbal | 29.870 | 71.086 |
| 131 | Mewa Khan | 29.819 | 70.511 | 181 | M. Rafique | 29.800 | 71.082 |
| 132 | Faqir Muhammad | 29.851 | 70.501 | 182 | M. Shafih | 29.545 | 71.021 |
| 133 | Bashir Ahmed | 30.422 | 70.738 | 183 | Irshad Hussain | 29.539 | 71.016 |
| 134 | Allah Nawaz | 30.422 | 70.740 | 184 | M. Younas | 29.539 | 71.014 |
| 135 | Sajjad Hussain | 30.558 | 70.691 | 185 | Faiz Akbar | 29.546 | 71.015 |
| 136 | M. Ramzan | 30.569 | 70.691 | 186 | M. Yameen Khan | 29.525 | 70.815 |
| 137 | Ghulam Muhammad | 30.569 | 70.691 | 187 | Haji Ghulam Yaseen | 29.549 | 70.809 |
| 138 | Jind Wada | 30.571 | 70.690 | 188 | Habib ur Rehman | 29.549 | 70.809 |
| 139 | Abdul Sattar | 30.611 | 70.681 | 189 | Zameer Hussain | 29.517 | 70.783 |
| 140 | Saifullah | 30.661 | 70.664 | 190 | Ch. Shafique | 29.403 | 70.917 |
| 141 | Atta ullah | 31.004 | 70.679 | 191 | Abdul Majeed | 29.399 | 70.900 |
| 142 | Umar Khan | 31.035 | 70.620 | 192 | Rana M. Yameen | 29.396 | 70.910 |
| 143 | Asif Hussain | 31.043 | 70.639 | 193 | Javed Khan | 29.407 | 70.895 |
| 144 | Allah Dita | 31.037 | 70.666 | 194 | Ch. M. Khan | 29.407 | 70.896 |
| 145 | M. Ibrahim | 30.917 | 70.689 | 195 | Malik Zaffar | 29.364 | 70.883 |
| 146 | Abdul Hameed | 30.920 | 70.681 | 196 | M. Sajid | 29.362 | 70.879 |
| 147 | Ghulam Fareed | 29.758 | 70.609 | 197 | Khizar Hayat | 29.323 | 70.799 |
| 148 | Malik Sohara Arain | 29.724 | 70.608 | 198 | Mahar M. Hafeez | 29.320 | 70.796 |
| 149 | Nabib Khan | 29.702 | 70.606 | 199 | Haji Ghulam Shabbir | 20.305 | 70.789 |
| 150 | Zaffar Iqbal | 29.702 | 70.606 | 200 | Ulfat Hussain | 20.290 | 70.781 |
| 201 | Khadim Hussain | 20.292 | 70.782 | 251 | M. Mumtaz | 29.842 | 71.725 |
| 202 | Saif ullah Khan | 30.517 | 71.562 | 252 | Adnan Haider | 29.843 | 71.724 |
| 203 | Allah Dita | 30.522 | 71.571 | 253 | Yasir Jan | 29.756 | 71.871 |
| 204 | M. Qasim | 30.576 | 71.045 | 254 | Iqbal Husnain Khan | 29.710 | 71.924 |
| 205 | M. Hanif | 30.576 | 71.052 | 255 | Muhammad Saleem | 31.436 | 74.232 |
| 206 | Qamar Javed | 30.580 | 71.156 | 256 | M. Ijaz | 29.981 | 71.424 |
| 207 | Allah Rakha | 30.058 | 71.156 | 257 | Mushtaq Ahmed | 29.985 | 71.428 |
| 208 | Ghulam Hassan | 30.576 | 71.031 | 258 | M. Sardar | 29.983 | 71.430 |
| 209 | Rana Liaquat | 30.570 | 70.968 | 259 | Jam Allah Wasaya | 30.106 | 71.627 |
| 210 | M. Arshad | 30.076 | 71.050 | 260 | Muhammad Asif | 30.084 | 71.653 |
| 211 | Waqas Ahmed | 30.853 | 70.940 | 261 | Shah Mehmood Qureshi | 30.067 | 71.634 |
| 212 | Khizar Mehtab | 30.860 | 70.940 | 262 | Altaf Hussain | 29.971 | 71.716 |
| 213 | Ijaz Hussain | 30.847 | 70.940 | 263 | Mazhar Abbas | 30.247 | 71.722 |
| 214 | Ghulam Rasool | 30.857 | 70.950 | 264 | Malik Mushtaq | 30.293 | 71.662 |
| 215 | M. Irfan | 30.946 | 71.641 | 265 | M. Imran | 30.286 | 71.679 |
| 216 | M.Rizawan | 30.946 | 71.065 | 266 | M. Farooq | 30.392 | 71.584 |
| 217 | M. Ajmal | 30.967 | 71.034 | 267 | M. Tariq | 30.362 | 71.544 |
| 218 | Rizwan | 30.963 | 71.039 | 268 | Mumtaz Hussain | 30.399 | 71.513 |
| 219 | Bashir Ahmed | 31.178 | 70.967 | 269 | Jaffar Iqbal | 30.181 | 71.445 |
| 220 | Tariq Mehmood | 31.196 | 71.002 | 270 | Zaffar Iqbal | 29.950 | 71.431 |
| 221 | Maqsood Ahmed | 31.166 | 71.174 | 271 | M. Anwar | 30.031 | 71.340 |
| 222 | M. Iqbal | 31.184 | 71.182 | 272 | Imam Baksh | 30.095 | 71.517 |
| 223 | Yasir Ghafoor | 30.803 | 71.246 | 273 | M. Fiaz | 30.254 | 71.558 |
| 224 | Ghulam Shabbir | 30.841 | 71.223 | 274 | Malik Akhtar | 30.097 | 71.330 |
| 225 | M. Zubair Alvi | 30.942 | 71.090 | 275 | Rana Saeed Ahmed | 29.963 | 71.356 |
| 226 | M. Hussain | 30.982 | 71.091 | 276 | Allah Ditta | 29.941 | 71.330 |
| 227 | Waqas Manzoor | 30.985 | 70.966 | 277 | Hussain Abbas | 29.601 | 71.239 |
| 228 | Altaf Hussain | 30.967 | 70.958 | 278 | Farhan Kareem | 29.561 | 71.233 |
| 229 | Waris Ali | 30.965 | 71.307 | 279 | CRI, Multan | 30.141 | 71.442 |
| 230 | Imran Satta | 30.908 | 71.295 | 280 | CRI, Multan | 30.141 | 71.442 |
| 231 | M. Asif | 29.787 | 71.555 | 281 | CRI, Multan | 30.141 | 71.442 |
| 232 | M. Javed | 29.786 | 71.558 | 282 | CRI, Multan | 30.141 | 71.442 |
| 233 | Haji M. Hanif | 29.783 | 71.514 | 283 | Shakeel Ahmed | 29.756 | 71.240 |
| 234 | Inam Ullah | 29.756 | 71.529 | 284 | Munir Ahmed | 29.699 | 71.174 |
| 235 | M. Afzal | 29.592 | 71.615 | 285 | M. Hussain | 30.457 | 72.313 |
| 236 | M. Shafiq | 29.578 | 71.529 | 286 | Wasem Ali | 30.454 | 72.316 |
| 237 | Rana M. Nadeem | 29.550 | 71.626 | 287 | Atiq-ur-Rehman | 30.418 | 72.292 |
| 238 | Akram Shah | 29.567 | 71.658 | 288 | Mehbob Alam | 30.388 | 72.361 |
| 239 | M. Waheed Ahmed | 29.494 | 71.506 | 289 | M. Idress | 30.494 | 72.274 |
| 240 | Zahid Rasool | 29.687 | 71.667 | 290 | Mohsin Ali | 30.473 | 72.307 |
| 241 | Ch. M. Riaz | 29.621 | 71.675 | 291 | Mushtaq Ahmed | 30.507 | 72.417 |
| 242 | M. Asghar Javed | 29.478 | 71.604 | 292 | M. Sajjad | 30.472 | 72.182 |
| 243 | Falak Sher | 29.555 | 71.431 | 293 | Mushtaq Ahmed | 30.519 | 72.247 |
| 244 | M. Tariq | 29.581 | 71.748 | 294 | Waseem Sajid | 30.519 | 72.331 |
| 245 | Iqbal Anjum | 29.687 | 71.667 | 295 | Zulfiqar Ali | 30.177 | 72.232 |
| 246 | Zaffar Iqbal | 29.684 | 71.916 | 296 | M. Fiaz Bashir | 30.175 | 72.120 |
| 247 | Zia M. | 29.681 | 71.860 | 297 | Punjab Seed Corperation | 30.328 | 72.031 |
| 248 | Malik Hashim | 29.644 | 71.916 | 298 | Punjab Seed Corperation | 30.328 | 72.031 |
| 249 | M. Sajjad | 29.692 | 71.934 | 299 | Punjab Seed Corperation | 30.328 | 72.031 |
| 250 | Dr. M. Akbar | 29.645 | 71.915 | 300 | Punjab Seed Corperation | 30.328 | 72.031 |
| 301 | Javed Iqbal | 30.435 | 72.132 | 351 | Hafiz Ghulam Mustafa | 30.045 | 72.096 |
| 302 | M. Bashir | 30.341 | 72.122 | 352 | M. Amir | 30.046 | 72.062 |
| 303 | M. Fazal | 30.304 | 72.173 | 353 | M. Ijaz | 29.944 | 71.044 |
| 304 | Raja Hassan Akhtar | 30.304 | 72.173 | 354 | Abdul Rehman | 29.934 | 71.961 |
| 305 | Tanveer Abbas | 30.223 | 71.957 | 355 | Ch. Tahir Mehmmod | 29.865 | 72.860 |
| 306 | Karam Abbas | 30.238 | 71.904 | 356 | Ch. Amir Mehmood | 29.860 | 72.083 |
| 307 | M. Ishfaq | 30.239 | 71.903 | 357 | Qaiser Abbas | 29.843 | 72.227 |
| 308 | Allah Yar | 30.228 | 71.885 | 358 | M. Amin | 29.810 | 72.213 |
| 309 | M. Adnan Hassan | 30.075 | 71.776 | 359 | Mumtaz Ahmed | 29.741 | 72.225 |
| 310 | Anwar Hussain Shah | 30.073 | 71.778 | 360 | M. Akram | 29.823 | 72.239 |
| 311 | M. Ismail | 30.044 | 71.761 | 361 | Rana Iqrar Hussain | 30.990 | 72.966 |
| 312 | M. Mazhar | 30.047 | 71.754 | 362 | Nisar Ahmed | 30.980 | 72.956 |
| 313 | Bakar Hussain | 30.358 | 71.875 | 363 | Allah Rakha | 30.213 | 73.062 |
| 314 | Akbar Hayat | 30.351 | 71.852 | 364 | Choudhary Nisar | 30.198 | 73.056 |
| 315 | Mahar Khadim | 30.353 | 71.872 | 365 | Akram Gujjar | 30.165 | 73.056 |
| 316 | M. Hayat | 30.351 | 71.852 | 366 | Muhammad Riaz | 30.171 | 73.056 |
| 317 | Zaffar Iqbal | 30.409 | 71.755 | 367 | Irfan Ahmed | 30.177 | 73.064 |
| 318 | Munir Ahmed Khan | 30.406 | 71.762 | 368 | Ch. M. Saleem | 30.364 | 73.028 |
| 319 | M. Ashraf | 30.442 | 71.798 | 369 | Nasir Ahmed | 30.370 | 73.026 |
| 320 | Gul Sher Khan | 30.442 | 71.797 | 370 | Maqsood Ahmed | 30.364 | 73.029 |
| 321 | M. Shafiq | 30.437 | 71.824 | 371 | Azhar Ali | 30.489 | 72.648 |
| 322 | M. Ijaz | 30.590 | 71.965 | 372 | Allah Yar | 30.471 | 72.631 |
| 323 | Ghulzar Hussain | 30.599 | 71.989 | 373 | M. Saad | 30.471 | 72.631 |
| 324 | Abdul Rasheed | 30.571 | 72.045 | 374 | Abdul Ghaffar | 30.478 | 72.631 |
| 325 | M. Jamil | 30.602 | 72.065 | 375 | Abdul Razzaq | 30.471 | 62.631 |
| 326 | M. Shafqat | 30.602 | 72.065 | 376 | Rana Abdul Ghaffar | 30.478 | 72.652 |
| 327 | Irfan Ali | 30.230 | 27.741 | 377 | M. Iftikhar | 30.434 | 72.628 |
| 328 | Rashid Ahmed | 30.230 | 72.741 | 378 | M. Idress | 30.425 | 72.670 |
| 329 | Arshad Ali | 30.220 | 72.840 | 379 | M. Azam | 30.447 | 72.663 |
| 330 | Khaliq Hussain | 30.178 | 72.753 | 380 | M. Hussain | 30.523 | 72.704 |
| 331 | M. Luqman | 30.114 | 72.815 | 381 | M. Haroon | 30.447 | 72.663 |
| 332 | M. Nadeem | 30.114 | 72.817 | 382 | Shahid Iqbal | 30.569 | 72.690 |
| 333 | Haji M. Iqbal | 30.015 | 72.790 | 383 | M. Habaib | 30.568 | 72.691 |
| 334 | M. N awaz | 30.075 | 72.691 | 384 | Niaz Ahmed | 30.587 | 72.752 |
| 335 | Haji M. Iqbal | 30.125 | 72.633 | 385 | Haji Shair | 31.022 | 73.121 |
| 336 | M. N awaz | 30.124 | 72.613 | 386 | Rai Umar Hayat | 31.011 | 73.113 |
| 337 | M. Kashif Ramay | 30.203 | 72.660 | 387 | Ghulam Khubaib | 30.987 | 73.179 |
| 338 | Liaquat Ali | 30.197 | 72.599 | 388 | Rizwan Zaffar | 30.977 | 73.208 |
| 339 | Abdul Kareem | 30.047 | 72.351 | 389 | M. Shafiq | 30.945 | 73.028 |
| 340 | Shabir Ahmed | 30.136 | 72.314 | 390 | M. Ali | 30.949 | 73.010 |
| 341 | M. Zaffar Iqbal | 30.060 | 72.328 | 391 | Aman Ullah | 30.876 | 72.927 |
| 342 | Fisal Shahzad | 30.057 | 72.321 | 392 | M. Asghar | 30.858 | 72.915 |
| 343 | Imdad Hussain | 30.083 | 72.373 | 393 | Abdul Qayoom | 30.815 | 72.826 |
| 344 | Riaz Ahmed | 30.074 | 72.364 | 394 | M. Anwar | 30.822 | 72.893 |
| 345 | M. Hanif | 30.164 | 72.480 | 395 | Muneer Ahmed | 30.844 | 72.751 |
| 346 | M. Ramzan | 30.162 | 72.484 | 396 | Danish | 30.899 | 72.728 |
| 347 | Malik Abdul Hameed | 29.982 | 72.486 | 397 | Abdul Jabbar | 30.434 | 72.628 |
| 348 | M. Azeem | 25.984 | 72.486 | 398 | Javed Riaz | 30.478 | 72.652 |
| 349 | Mian Mukhtar Ahmed | 29.986 | 72.532 | 399 | Amir Ghaffar | 30.471 | 72.631 |
| 350 | Nasir Ali | 29.950 | 72.662 | 400 | Atta Ullah | 30.435 | 72.663 |

# 2.1 Supplementary Tables:

**Supplementary Table 3. District wise ranking of average Cry1Ac expression and insect mortality% in the Upper Canopy.**

| District | Cry1Ac Expression (µg g-1) | Insect Mortality % |
| --- | --- | --- |
| Muzaffargarh | 2.12 | 86 |
| Khanewal | 2.09 | 86 |
| DG Khan | 2.09 | 77 |
| Vehari | 2.00 | 86 |
| Lodhran | 1.94 | 83 |
| Rajanpur | 1.84 | 84 |
| Pakpattan | 1.83 | 82 |
| Multan | 1.67 | 79 |
| Sahiwal | 1.64 | 80 |
| Bahawalnagar | 1.56 | 80 |
| Rahim Yar Khan | 1.54 | 81 |
| Bahawalpur | 1.50 | 76 |
| Faisalabad | 1.42 | 74 |
| Toba Tek Singh | 1.37 | 71 |
| Layyah | 1.32 | 69 |

**Supplementary Table 4. District wise ranking of average Cry1Ac expression and insect mortality% in the Middle Canopy.**

| District | Cry1Ac Expression (µg g-1) | Insect Mortality % |
| --- | --- | --- |
| DG Khan | 1.06 | 61 |
| Khanewal | 0.92 | 60 |
| Bahawalpur | 0.91 | 58 |
| Muzaffargarh | 0.91 | 58 |
| Bahawalnagar | 0.81 | 55 |
| Rahim Yar Khan | 0.77 | 51 |
| Multan | 0.77 | 52 |
| Lodhran | 0.75 | 54 |
| Vehari | 0.64 | 46 |
| Rajanpur | 0.64 | 51 |
| Toba Tek Singh | 0.59 | 42 |
| Pakpattan | 0.55 | 44 |
| Sahiwal | 0.51 | 35 |
| Faisalabad | 0.50 | 38 |
| Layyah | 0.31 | 21 |

**Supplementary Table 5. District wise ranking of average Cry1Ac expression and insect mortality% in the Lower Canopy.**

| District | Cry1Ac Expression (µg g-1) | Insect Mortality % |
| --- | --- | --- |
| Bahawalnagar | 0.13 | 12 |
| Bahawalpur | 0.03 | 2 |
| DG Khan | 0.09 | 7 |
| Muzaffargarh | 0.09 | 6 |
| Rahim Yar Khan | 0.09 | 7 |
| Rajanpur | 0.06 | 4 |
| Multan | 0.06 | 4 |
| Khanewal | 0.05 | 3 |
| Vehari | 0.05 | 2 |
| Sahiwal | 0.03 | 2 |
| Pakpattan | 0.02 | 1 |
| Faisalabad | 0.00 | 0 |
| Layyah | 0.00 | 0 |
| Lodhran | 0.00 | 0 |
| Toba Tek Singh | 0.00 | 0 |

**Supplementary Table 6. District wise ranking of average Cry1Ac expression and insect mortality% in the Ten Days Bolls.**

| District | Cry1Ac Expression (µg g-1) | Insect Mortality % |
| --- | --- | --- |
| Layyah | 0.71 | 43 |
| Multan | 0.68 | 45 |
| Rajanpur | 0.62 | 42 |
| Rahim Yar Khan | 0.60 | 39 |
| DG Khan | 0.54 | 36 |
| Muzaffargarh | 0.52 | 35 |
| Faisalabad | 0.46 | 35 |
| Lodhran | 0.45 | 30 |
| Bahawalnagar | 0.43 | 31 |
| Khanewal | 0.43 | 31 |
| Pakpattan | 0.40 | 28 |
| Sahiwal | 0.38 | 28 |
| Vehari | 0.36 | 26 |
| Toba Tek Singh | 0.30 | 30 |
| Bahawalpur | 0.29 | 14 |

**Supplementary Table 7. District wise ranking of average Cry1Ac expression and insect mortality% in the Squares.**

| District | Cry1Ac Expression (µg g-1) | Insect Mortality % |
| --- | --- | --- |
| Rahim Yar Khan | 1.30 | 70 |
| DG Khan | 1.29 | 67 |
| Bahawalnagar | 1.03 | 61 |
| Multan | 0.89 | 58 |
| Khanewal | 0.85 | 56 |
| Bahawalpur | 0.82 | 56 |
| Rajanpur | 0.78 | 52 |
| Vehari | 0.78 | 53 |
| Lodhran | 0.75 | 50 |
| Pakpattan | 0.70 | 49 |
| Muzaffargarh | 0.70 | 50 |
| Sahiwal | 0.47 | 35 |
| Layyah | 0.45 | 32 |
| Toba Tek Singh | 0.39 | 32 |
| Faisalabad | 0.33 | 26 |

**Supplementary Table 8. Variety wise ranking of average Cry1Ac expression and insect mortality% in the Upper Canopy.**

| Variety | Expression | Mortality % | Observation recorded from how many districts |
| --- | --- | --- | --- |
| Jullundur Seeds | 2.65 | 90 | 1 |
| N-8 | 2.62 | 90 | 1 |
| BS-80 | 2.42 | 90 | 3 |
| BS-70 | 2.24 | 92 | 1 |
| FH-490 | 2.14 | 92 | 1 |
| IUB-13 | 2.07 | 84 | 14 |
| BS-18 | 2.01 | 86 | 13 |
| FH-142 | 1.87 | 81 | 12 |
| Cemb-33 | 1.84 | 86 | 1 |
| BS-15 | 1.71 | 81 | 12 |
| FH-Lalazar | 1.70 | 75 | 2 |
| NS-181 | 1.48 | 78 | 1 |
| FH-992 | 1.45 | 81 | 1 |
| MNH-1016 | 1.44 | 73 | 5 |
| S-2002 | 1.32 | 75 | 1 |
| SS-32 | 1.27 | 75 | 8 |
| MNH-886 | 1.18 | 71 | 3 |
| NIAB-878 | 1.17 | 72 | 3 |
| MNH-1020 | 1.06 | 70 | 1 |
| IUB-15 | 0.96 | 64 | 2 |
| VH-179 | 0.46 | 49 | 1 |
| Z-33 | 0.26 | 28 | 1 |
| NIAB-KIRAN | 0.00 | 0 | 1 |

**Supplementary Table 9. Variety wise ranking of average Cry1Ac expression and insect mortality% in the Middle Canopy.**

| Variety | Expression | Mortality % | Observation recorded from how many districts |
| --- | --- | --- | --- |
| BS-70 | 0.96 | 67 | 1 |
| Cemb-33 | 0.96 | 68 | 1 |
| Jullundur Seeds | 0.96 | 70 | 1 |
| BS-15 | 0.88 | 57 | 12 |
| BS-18 | 0.82 | 54 | 13 |
| FH-490 | 0.80 | 61 | 1 |
| FH-Lalazar | 0.80 | 57 | 2 |
| IUB-13 | 0.76 | 51 | 14 |
| FH-142 | 0.72 | 47 | 12 |
| MNH-1016 | 0.70 | 51 | 5 |
| SS-32 | 0.68 | 48 | 8 |
| BS-80 | 0.64 | 42 | 3 |
| FH-992 | 0.62 | 44 | 1 |
| N-8 | 0.54 | 46 | 1 |
| MNH-886 | 0.51 | 34 | 3 |
| MNH-1020 | 0.42 | 35 | 1 |
| NIAB-878 | 0.38 | 35 | 3 |
| NS-181 | 0.38 | 27 | 1 |
| IUB-15 | 0.28 | 21 | 2 |
| S-2002 | 0.28 | 17 | 1 |
| Z-33 | 0.22 | 23 | 1 |
| VH-179 | 0.20 | 13 | 1 |
| NIAB-KIRAN | 0.00 | 0 | 1 |

**Supplementary Table 10. Variety wise ranking of average Cry1Ac expression and insect mortality% in the lower Canopy.**

| Variety | Expression | Mortality % | Observation recorded from how many districts |
| --- | --- | --- | --- |
| Cemb-33 | 0.13 | 8 | 1 |
| BS-15 | 0.05 | 4 | 12 |
| NIAB-KIRAN | 0.00 | 0 | 1 |
| BS-18 | 0.00 | 0 | 13 |
| BS-70 | 0.00 | 0 | 1 |
| BS-80 | 0.00 | 0 | 3 |
| FH-142 | 0.00 | 0 | 12 |
| FH-490 | 0.00 | 0 | 1 |
| FH-992 | 0.00 | 0 | 1 |
| FH-Lalazar | 0.00 | 0 | 2 |
| IUB-13 | 0.00 | 0 | 14 |
| IUB-15 | 0.00 | 0 | 2 |
| Jullender Sesds | 0.00 | 0 | 1 |
| MNH-1016 | 0.00 | 0 | 5 |
| MNH-1020 | 0.00 | 0 | 1 |
| MNH-886 | 0.00 | 0 | 3 |
| N-8 | 0.00 | 0 | 1 |
| NIAB-878 | 0.00 | 0 | 3 |
| NS-181 | 0.00 | 0 | 1 |
| S-2002 | 0.00 | 0 | 1 |
| SS-32 | 0.00 | 0 | 8 |
| VH-179 | 0.00 | 0 | 1 |
| Z-33 | 0.00 | 0 | 1 |

**Supplementary Table 11. Variety wise ranking of average Cry1Ac expression and insect mortality% in the Ten Days Bolls.**

| Variety | Expression | Mortality % | Observation recorded from how many districts |
| --- | --- | --- | --- |
| BS-80 | 0.89 | 57 | 3 |
| FH-490 | 0.64 | 46 | 1 |
| Cemb-33 | 0.63 | 44 | 1 |
| FH-142 | 0.62 | 41 | 12 |
| IUB-13 | 0.59 | 37 | 14 |
| Jullundur Seeds | 0.54 | 40 | 1 |
| BS-15 | 0.49 | 37 | 12 |
| FH-Lalazar | 0.45 | 40 | 2 |
| NS-181 | 0.43 | 33 | 1 |
| SS-32 | 0.43 | 27 | 8 |
| N-8 | 0.42 | 33 | 1 |
| IUB-15 | 0.39 | 28 | 2 |
| MNH-886 | 0.38 | 30 | 3 |
| MNH-1020 | 0.38 | 41 | 1 |
| BS-18 | 0.38 | 25 | 13 |
| Z-33 | 0.36 | 24 | 1 |
| S-2002 | 0.32 | 40 | 1 |
| MNH-1016 | 0.31 | 21 | 5 |
| NIAB-878 | 0.23 | 16 | 3 |
| BS-70 | 0.20 | 13 | 1 |
| FH-992 | 0.20 | 15 | 1 |
| VH-179 | 0.18 | 11 | 1 |
| NIAB-KIRAN | 0.00 | 0 | 1 |

**Supplementary Table 12. Variety wise ranking of average Cry1Ac expression and insect mortality% in Squares.**

| Variety | Expression | Mortality % | Observation recorded from how many districts |
| --- | --- | --- | --- |
| Cemb-33 | 1.35 | 71 | 1 |
| N-8 | 1.22 | 74 | 1 |
| BS-70 | 1.18 | 64 | 1 |
| BS-18 | 0.89 | 58 | 13 |
| BS-80 | 0.89 | 58 | 3 |
| IUB-13 | 0.87 | 50 | 14 |
| FH-992 | 0.80 | 59 | 1 |
| BS-15 | 0.80 | 54 | 12 |
| NIAB-878 | 0.77 | 56 | 3 |
| FH-142 | 0.75 | 46 | 12 |
| SS-32 | 0.71 | 47 | 8 |
| MNH-1016 | 0.70 | 48 | 5 |
| FH-490 | 0.70 | 50 | 1 |
| MNH-886 | 0.66 | 41 | 3 |
| NS-181 | 0.62 | 46 | 1 |
| Z-33 | 0.56 | 46 | 1 |
| Jullundur Seeds | 0.48 | 38 | 1 |
| FH-Lalazar | 0.40 | 35 | 2 |
| S-2002 | 0.34 | 30 | 1 |
| IUB-15 | 0.33 | 29 | 2 |
| VH-179 | 0.32 | 39 | 1 |
| MNH-1020 | 0.24 | 15 | 1 |
| NIAB-KIRAN | 0.00 | 0 | 1 |

**Supplementary Table 13. Different inputs and Bt cotton expression from eight nine location across Punjab. Whereas NOI; number of irrigations, RF; rainfall, N; nitrogen fertilizer (Urea 50 Kg bags/acre), P; phosphorus (DAP 50 Kg bags/acre), K; potassium (Kgs/acre) and Micro; Micronutrients (Kg/acre).**

| Sr. No. | Expression | NOI | RF | N | P | K | Micro |
| --- | --- | --- | --- | --- | --- | --- | --- |
|  | 2.26 | 6 | 3 | 2.5 | 2 | 0 | 1 |
|  | 1.42 | 4 | 3 | 2 | 1.5 | 0 | 0 |
|  | 1.74 | 5 | 1 | 2 | 2 | 0 | 0 |
|  | 0.8 | 3 | 1 | 1 | 1 | 0 | 0 |
|  | 0.28 | 2 | 1 | 1 | 1 | 0 | 0 |
|  | 1.24 | 4 | 3 | 2 | 1.5 | 0 | 0 |
|  | 1.25 | 5 | 2 | 2.5 | 1.5 | 0 | 4 |
|  | 1.86 | 09 | 3 | 4 | 1 | 0 | 2 |
|  | 1.25 | 09 | 2 | 4 | 1 | 0 | 2 |
|  | 2.5 | 7 | 2 | 4 | 1 | 0 | 0 |
|  | 1.38 | 6 | 1 | 2 | 1 | 0 | 0 |
|  | 1.12 | 7 | 2 | 1.5 | 1 | 10 | 10 |
|  | 2.25 | 2 | 3 | 3 | 1 | 20 | 20 |
|  | 1.84 | 5 | 3 | 2 | 1.5 | 0 | 0 |
|  | 0.52 | 5 | 2 | 1 | 1 | 0 | 0 |
|  | 1.32 | 5 | 3 | 2 | 1.5 | 0 | 0 |
|  | 1.04 | 5 | 2 | 2 | 1.5 | 0 | 15 |
|  | 1.27 | 5 | 1 | 2 | 2 | 0 | 15 |
|  | 1.45 | 7 | 1 | 2 | 1 | 0 | 25 |
|  | 2.82 | 19 | 2 | 3 | 2 | 0 | 0 |
|  | 3.54 | 19 | 3 | 4 | 3 | 0 | 45 |
|  | 3.42 | 16 | 3 | 4 | 2.5 | 0 | 40 |
|  | 1.05 | 7 | 2 | 2 | 1 | 0 | 0 |
|  | 0.26 | 5 | 2 | 2 | 0 | 0 | 0 |
|  | 0.86 | 6 | 1 | 2 | 1 | 0 | 0 |
|  | 1.24 | 7 | 1 | 2 | 1 | 0 | 0 |
|  | 3.42 | 16 | 1 | 6 | 3 | 0 | 0 |
|  | 2.9 | 15 | 1 | 6 | 3 | 0 | 0 |
|  | 1.23 | 10 | 1 | 3 | 1 | 0 | 0 |
|  | 1.06 | 12 | 1 | 2 | 1 | 0 | 0 |
|  | 2.94 | 20 | 1 | 4 | 2 | 10 | 2 |
|  | 2.24 | 12 | 1 | 4 | 2 | 0 | 10 |
|  | 1.66 | 12 | 1 | 3 | 1.5 | 0 | 10 |
|  | 1.45 | 10 | 1 | 3 | 1.5 | 0 | 0 |
|  | 1.25 | 10 | 1 | 2 | 2 | 0 | 20 |
|  | 1.52 | 10 | 1 | 4 | 1.5 | 0 | 0 |
|  | 1.22 | 8 | 1 | 2.5 | 0.5 | 20 | 20 |
|  | 1.06 | 7 | 1 | 1.5 | 1 | 0 | 10 |
|  | 0.68 | 6 | 1 | 1.5 | 0.5 | 0 | 0 |
|  | 0.42 | 5 | 1 | 1.5 | 0 | 0 | 0 |
|  | 0.34 | 05 | 1 | 1.5 | 0 | 0 | 0 |
|  | 0.22 | 4 | 1 | 2 | 1 | 0 | 0 |
|  | 1.26 | 10 | 1 | 2 | 2 | 0 | 0 |
|  | 2.58 | 12 | 1 | 4 | 2 | 10 | 30 |
|  | 1.2 | 4 | 1 | 2 | 1 | 0 | 0 |
|  | 1.06 | 6 | 1 | 2 | 1 | 0 | 0 |
|  | 0.68 | 6 | 1 | 0 | 1 | 0 | 0 |
|  | 0 | 15 | 1 | 1 | 1 | 0 | 0 |
|  | 0.84 | 10 | 1 | 1.5 | 1 | 10 | 0 |
|  | 1.54 | 10 | 1 | 2.5 | 1 | 10 | 0 |
|  | 1.86 | 12 | 1 | 1.5 | 1 | 0 | 0 |
|  | 1.92 | 12 | 1 | 2 | 1 | 15 | 15 |
|  | 1.22 | 10 | 1 | 2 | 1 | 0 | 0 |
|  | 1.24 | 10 | 1 | 2 | 1 | 0 | 0 |
|  | 1 | 8 | 1 | 1 | 0 | 0 | 0 |
|  | 1.24 | 8 | 1 | 2 | 1 | 0 | 0 |
|  | 2.06 | 10 | 2 | 2.5 | 2 | 0 | 20 |
|  | 1.86 | 10 | 2 | 3 | 1.5 | 0 | 15 |
|  | 2.3 | 13 | 2 | 3 | 2 | 0 | 20 |
|  | 1.24 | 13 | 2 | 2 | 1 | 0 | 20 |
|  | 1.35 | 10 | 1 | 2 | 1.5 | 0 | 15 |
|  | 1.48 | 15 | 1 | 3 | 1 | 0 | 15 |
|  | 1.62 | 16 | 1 | 2 | 2 | 1 | 20 |
|  | 2.14 | 20 | 1 | 3 | 1.5 | 0 | 0 |
|  | 2.36 | 30 | 1 | 4 | 2 | 0 | 30 |
|  | 1.44 | 25 | 1 | 3 | 2 | 0 | 0 |
|  | 0.56 | 12 | 1 | 2 | 1 | 0 | 0 |
|  | 1.24 | 12 | 1 | 3 | 2 | 0 | 0 |
|  | 1.45 | 20 | 1 | 3 | 2 | 0 | 0 |
|  | 1.65 | 2 | 1 | 3 | 2 | 0 | 0 |
|  | 2.62 | 10 | 1 | 4 | 2.5 | 0 | 0 |
|  | 1.45 | 16 | 1 | 5 | 1 | 0 | 0 |
|  | 1.06 | 15 | 1 | 4 | 2 | 0 | 0 |
|  | 1.45 | 15 | 2 | 6 | 1 | 0 | 0 |
|  | 1.36 | 14 | 1 | 3 | 1 | 0 | 0 |
|  | 1.48 | 14 | 1 | 3 | 1 | 0 | 0 |
|  | 0.68 | 10 | 1 | 1 | 1 | 0 | 0 |
|  | 1.82 | 17 | 1 | 3 | 2 | 0 | 20 |
|  | 0.84 | 12 | 2 | 1.5 | 1.5 | 0 | 20 |
|  | 0.85 | 16 | 2 | 2 | 1 | 0 | 0 |
|  | 1.25 | 15 | 2 | 2 | 0.5 | 0 | 0 |
|  | 1.23 | 16 | 2 | 2 | 2 | 0 | 25 |
|  | 1.02 | 14 | 2 | 2 | 1.5 | 0 | 10 |
|  | 1.32 | 18 | 2 | 2.5 | 0.5 | 0 | 0 |
|  | 1.2 | 18 | 2 | 1 | 0.5 | 0 | 0 |
|  | 0.56 | 18 | 2 | 0.5 | 0.5 | 0 | 0 |
|  | 1.56 | 18 | 2 | 2 | 2 | 0 | 0 |
|  | 1.06 | 17 | 2 | 2 | 1 | 0 | 20 |
|  | 0.46 | 17 | 2 | 0.5 | 0.5 | 0 | 20 |
